# Hepatocyte‐Specific β‐Catenin Deletion During Severe Liver Injury Provokes Cholangiocytes to Differentiate Into Hepatocytes

**DOI:** 10.1002/hep.30270

**Published:** 2019-01-04

**Authors:** Jacquelyn O. Russell, Wei‐Yu Lu, Hirohisa Okabe, Marc Abrams, Michael Oertel, Minakshi Poddar, Sucha Singh, Stuart J. Forbes, Satdarshan P. Monga

**Affiliations:** ^1^ Department of Pathology University of Pittsburgh Pittsburgh PA; ^2^ Pittsburgh Liver Research Center University of Pittsburgh and University of Pittsburgh Medical Center Pittsburgh PA; ^3^ MRC Centre for Regenerative Medicine University of Edinburgh Edinburgh UK; ^4^ Department of Medicine University of Pittsburgh School of Medicine and University of Pittsburgh Medical Center Pittsburgh PA; ^5^ Department of Gastroenterological Surgery Kumamoto University Kumamoto Japan; ^6^ Dicerna Pharmaceuticals Boston MA; ^7^ Centre for Liver Research University of Birmingham Birmingham UK

## Abstract

Liver regeneration after injury is normally mediated by proliferation of hepatocytes, although recent studies have suggested biliary epithelial cells (BECs) can differentiate into hepatocytes during severe liver injury when hepatocyte proliferation is impaired. We investigated the effect of hepatocyte‐specific β‐catenin deletion in recovery from severe liver injury and BEC‐to‐hepatocyte differentiation. To induce liver injury, we administered choline‐deficient, ethionine‐supplemented (CDE) diet to three different mouse models, the first being mice with deletion of β‐catenin in both BECs and hepatocytes (*Albumin‐Cre; Ctnnb1^flox/flox^* mice). In our second model, we performed hepatocyte lineage tracing by injecting *Ctnnb1^flox/flox^*; *Rosa‐stop^flox/flox^‐EYFP* mice with the adeno‐associated virus serotype 8 encoding Cre recombinase under the control of the thyroid binding globulin promoter, a virus that infects only hepatocytes. Finally, we performed BEC lineage tracing via *Krt19‐Cre^ERT^*; *Rosa‐stop^flox/flox^‐tdTomato *mice. To observe BEC‐to‐hepatocyte differentiation, mice were allowed to recover on normal diet following CDE diet–induced liver injury. Livers were collected from all mice and analyzed by quantitative real‐time polymerase chain reaction, western blotting, immunohistochemistry, and immunofluorescence. We show that mice with lack of β‐catenin in hepatocytes placed on the CDE diet develop severe liver injury with impaired hepatocyte proliferation, creating a stimulus for BECs to differentiate into hepatocytes. In particular, we use both hepatocyte and BEC lineage tracing to show that BECs differentiate into hepatocytes, which go on to repopulate the liver during long‐term recovery. *Conclusion*: β‐catenin is important for liver regeneration after CDE diet–induced liver injury, and BEC‐derived hepatocytes can permanently incorporate into the liver parenchyma to mediate liver regeneration.

**Abbreviations**
AAV8‐TBG‐Cre: adeno‐associated virus serotype 8 encoding Cre recombinase under the hepatocyte‐specific thyroid binding globulin promoter
ALP: alkaline phosphatase
ALT: alanine aminotransferase
ANOVA: analysis of variance
AST: aspartate aminotransferase
BECs: biliary epithelial cells
CDE: choline‐deficient, ethionine‐supplemented
EpCAM: epithelial cell adhesion molecule
EYFP: enhanced yellow fluorescence protein
GS: glutamine synthetase
IHC: immunohistochemistry
KO: knockout
LR: liver regeneration
PBS: phosphate‐buffered saline
WT: wild‐type

Despite the liver's capacity for regeneration, chronic liver disease and cirrhosis is the twelfth leading cause of death in the United States.^1^ This significant morbidity is attributable to lack of treatments for advanced liver disease besides liver transplantation, for which there is a severe shortage of donor organs.^2^ Often, hepatocytes and biliary epithelial cells (BECs) can replicate to replenish their respective cell types and eventually restore hepatic mass following injury. However, livers of patients with chronic liver disease exhibit ductular reaction,^3^, ^4^, ^5^ with the degree of BEC expansion correlating with disease severity.^6^, ^7^ The role of ductular reaction in liver regeneration (LR) remains controversial, although studies are beginning to show BECs may be giving rise to hepatocytes. Indeed, experimentally, when endogenous hepatocyte proliferation is impaired, reactive BECs^8^, ^9^ can differentiate into hepatocytes to mediate repair.^10^, ^11^ Alternatively, hepatocytes can differentiate into BECs, which can then be incorporated into biliary ductules^12^, ^13^ and may revert back to hepatocytes when injury is withdrawn.^14^ There is evidence of BEC‐to‐hepatocyte differentiation in both humans^15^, ^16^ and animal models of liver injury where hepatocyte proliferation is blocked^17^, ^18^, ^19^ or after near total loss of hepatocytes.^20^

The choline‐deficient, ethionine‐supplemented (CDE) diet is a well‐known liver injury diet that induces proliferation of reactive BECs.^21^, ^22^ Recent lineage tracing studies have demonstrated limited contribution of BECs to hepatocytes in CDE diet–fed mice,^23^, ^24^, ^25^ leading to the conclusion that BECs do not contribute significantly to the restoration of hepatocyte mass after chronic liver injury. However, the CDE diet does not block hepatocyte proliferation^25^ and thus is unable to provide the correct milieu for BEC‐to‐hepatocyte transdifferentiation. Because the Wnt/β‐catenin signaling pathway is a major driver of hepatocyte proliferation during LR,^26^, ^27^ we investigated if CDE diet–induced injury to conditional β‐catenin knockout mice will necessitate BEC‐mediated liver repair. We used multiple mouse models to perform hepatocyte and BEC lineage tracing in mice that underwent modulation of β‐catenin expression and were administered CDE diet. Our results demonstrate that loss of β‐catenin in hepatocytes during CDE diet–induced liver injury indeed impairs hepatocyte proliferation, triggering the expansion of BECs, which subsequently differentiate into hepatocytes. We have successfully established a model that will lend itself well to the study of the mechanisms of BEC expansion and differentiation.

## Materials and Methods

### MOUSE STRAINS, VIRAL INFECTIONS, TAMOXIFEN ADMINISTRATION, *IN VIVO* RNAi, AND DIET

All animals are housed in temperature‐ and light‐controlled facilities and are maintained in accordance with the Guide for Care and Use of Laboratory Animals and the Animal Welfare Act. *Albumin‐Cre;Ctnnb1^flox/flox^* mice or knockout (KO) 1 and wild‐type (WT) 1 littermate controls^28^ were maintained on a C57BL/6 background. *Ctnnb1^flox/flox^*; *Rosa‐stop^flox/flox^‐EYFP *reporter mice were generated through breeding *Ctnnb1^flox/flox^* mice with *Rosa‐stop^flox/flox^‐EYFP* mice (Jackson Laboratories). *Krt19‐Cre^ERT^*; *Rosa‐stop^flox/flox^‐tdTomato *reporter mice were described previously.^17^ To label hepatocytes and generate KO2 mice, 23‐25‐day‐old *Ctnnb1^flox/flox^*; *Rosa‐stop^flox/flox^‐EYFP *mice were injected intraperitoneally with 1×10^12^ genome copies of adeno‐associated virus serotype 8 encoding Cre recombinase under the hepatocyte‐specific thyroid binding globulin promoter (AAV8‐TBG‐Cre) (Penn Vector Core) followed by a 12‐day washout period. To generate WT2 mice, the same AAV8‐TBG‐Cre was injected into or *Ctnnb1^+/+^*; *Rosa‐stop^flox/flox^‐EYFP *mice. To label BECs, *Krt19‐Cre^ERT^*; *Rosa‐stop^flox/flox^‐tdTomato *mice were given 3 doses of 12.5 mg/kg tamoxifen during postnatal week 1, followed by 2 weeks of washout. For the liver injury time point, 4‐5‐week‐old mice were given choline‐deficient diet (Envigo Teklad Diets) supplemented with 0.15% ethionine drinking water (Acros Organics, 146170100) for 2‐3 weeks (*Krt19‐Cre^ERT^*; *Rosa‐stop^flox/flox^‐tdTomato* mice). For recovery time points, animals were switched back to normal chow diet for 3 days up to 6 months after 2 weeks of CDE diet. For *in vivo* knockdown of β‐catenin expression, both C57BL6/NJ (Charles River) and *Krt19‐Cre^ERT^*; *Rosa‐stop^flox/flox^‐tdTomato* mice were given biweekly or weekly subcutaneous injections of 5 mg/kg C*tnnb1*‐siRNA dissolved in phosphate‐buffered saline (PBS) or PBS as a control starting 1 week prior to CDE diet administration and continuing throughout CDE diet and recovery periods. The small interfering RNA (siRNA) (Dicerna Pharmaceuticals) was conjugated to N‐acetylgalactosamine, which allows hepatocyte‐specific knockdown of target gene expression.^29^ Serum biochemistry analysis was performed by automated methods at the University of Pittsburgh Medical Center clinical chemistry laboratory. All studies were performed according to the guidelines of the National Institutes of Health and the University of Pittsburgh Institutional Animal Use and Care Committee.

### IMMUNOHISTOCHEMISTRY

See Supporting Methods.

### IMMUNOFLUORESCENCE

See Supporting Methods.

### CELL QUANTIFICATION

In the negative lineage tracing model, samples were triple stained for β‐catenin**, **enhanced yellow fluorescence protein (EYFP), and Hnf4α as described above. For each sample, eight images at ×200 magnification of periportal regions were taken and in each image the number of EYFP+/Hnf4α+ and EYFP‐/Hnf4α+ cells were counted. A minimum of 700 cells were counted for every sample. In the positive lineage tracing model, images were obtained on a Zeiss Axiovert 200 microscope using a Zeiss Axiocam MR camera. Cell counts were performed manually on blinded slides and more than 20 consecutive fields at ×200 magnification. Hepatocytes and cholangiocytes were defined as Hnf4α and CK19 positive cells, respectively. Cells were counted with ImageJ cell counter program.

### WESTERN BLOTTING

See Supporting Methods.

### RT‐PCR

See Supporting Methods.

### STATISTICS

For analysis of serum biochemistry between two groups, a two‐tailed *t* test was performed. For analysis of gene expression data between more than two groups, a one‐way analysis of variance (ANOVA) was performed. For analysis of cell counts, such as proliferating hepatocytes, a Mann‐Whitney U test was performed. For analysis of change in body weight over time, a two‐way ANOVA was performed. A *P* < 0.05 was considered significant, and plots are mean ± SD. All statistical analysis and graph generation was performed using GraphPad Prism 7 software.

## Results

### β‐CATENIN IS IMPORTANT FOR LR AFTER CDE DIET–INDUCED LIVER INJURY

**Figure 1:**
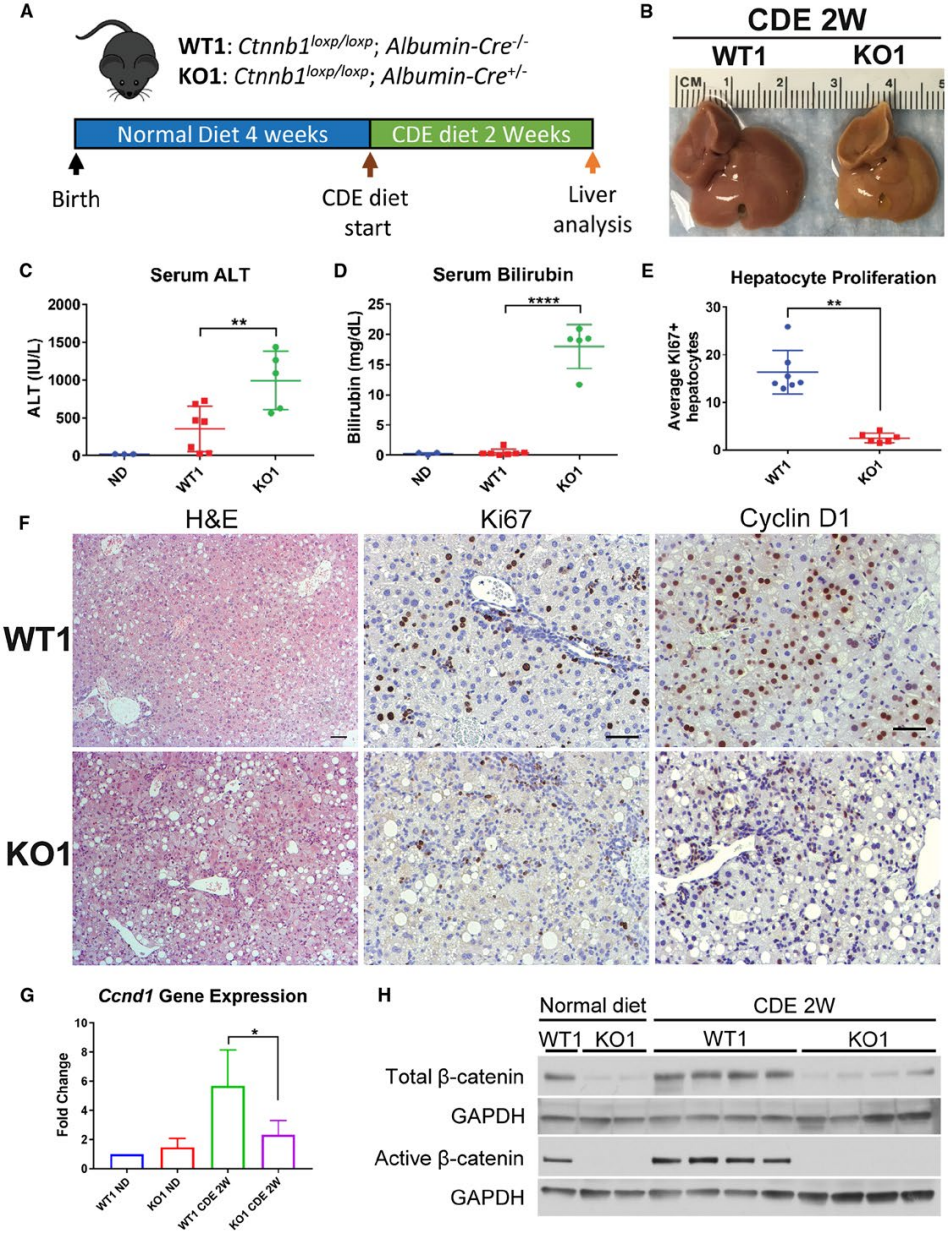
KO1 mice display severe liver injury and impaired hepatocyte proliferation after CDE diet. (A) Four‐week‐old male WT1 and KO1 mice were placed on CDE diet for 2W to induce liver injury. (B) Gross liver morphology reveals small and pale livers in KO1 mice after 2W of CDE diet. (C) Serum liver injury marker ALT is significantly elevated in KO1 mice after CDE diet compared with both WT1 mice and baseline ND levels (*t* test, ** = *P* < 0.01). (D) Serum bilirubin is significantly elevated in KO1 mice after CDE diet compared with both WT1 mice and baseline ND levels (*t* test, **** = *P* < 0.0001). (E) Quantification of hepatocyte proliferation in mice after 2W of CDE diet reveals significantly impaired hepatocyte proliferation in KO1 mice (Mann‐Whitney U test, ** = *P* < 0.01). (F) KO1 mice display prominent steatohepatitis and few Ki67 or Cyclin D1‐positive hepatocytes (scale bar 50 µm). (G) Hepatic *Ccnd1* gene expression is upregulated after 2W of CDE diet, but is impaired in KO1 mice (one‐way ANOVA, * = *P* < 0.05). (H) Whole liver lysates display dramatically reduced levels of β‐catenin and active β‐catenin in KO1 mice, whereas WT1 animals exhibit upregulation of both proteins after CDE diet. Abbreviations: ND, normal diet; W, weeks.

To test whether lack of β‐catenin in hepatocytes impairs hepatocyte proliferation in a chronic liver injury setting, we placed KO1 mice^28^ lacking β‐catenin in hepatocytes and BECs, and WT1 mice on CDE diet for 2 weeks (Fig. 1A). KO1 showed severe histological abnormalities including steatohepatitis (Fig. 1F), with gross liver morphology displaying pale and smller livers (Fig. 1B). Serum liver injury markers, alanine aminotransferase (ALT) (Fig. 1C), serum aspartate aminotransferase (AST) (Supporting Fig. S1D), and bilirubin (Fig. 1D) were significantly elevated in KO1 compared with WT1 on CDE diet. There was no difference in serum alkaline phosphatase (ALP) levels in WT1 and KO1 mice (Supporting Fig. S1E). Both WT1 and KO1 displayed characteristic expansion of reactive BECs, which are positive for BEC‐marker CK19 (Supporting Fig. S1A), although KO1 displayed a more robust BEC expansion as determined by increased expression of BEC markers *Krt19* and *Sox9* (Supporting Fig. S1F,G). KO1 also displayed increased fibrosis by Sirius Red staining (Supporting Fig. S1A) and increased expression of profibrotic genes *Col1a1* (Supporting Fig. S1B) and *Acta2* (Supporting Fig. S1C,H). We detected increased cell death as evidenced by terminal deoxynucleotidyl transferase–mediated deoxyuridine triphosphate nick‐end labeling staining (Supporting Fig. S1A) and increased hepatocyte senescence in KO1 as evidenced by an increase in p21‐positive hepatocytes (Supporting Fig. S1A) and increased levels of p21 protein (Supporting Fig. S1H) compared with WT1. Collectively, we detect severe liver injury in KO1 mice on CDE diet.

To determine if there was a defect in LR in KO1, we assessed hepatocyte proliferation via Ki67 and Cyclin D1 immunohistochemistry (IHC) (Fig. 1F). Whereas WT1 displayed robust hepatocyte proliferation and Cyclin D1 expression especially in periportal hepatocytes, KO1 displayed significantly impaired hepatocyte proliferation (Fig. 1E) and reduced *Ccnd1* expression (Fig. 1G) and protein (Supporting Fig. S1H). As expected, low levels of β‐catenin protein were observed in KO1 livers, likely due to nonparenchymal cells (Fig. 1H). However, there was no expression of active‐β‐catenin (hypophosphorylated) in KO1 mice even after CDE diet (Fig. 1H). Alternatively, there was increased total and active‐β‐catenin levels in WT1 mice on CDE diet compared with normal diet (Fig. 1H). This suggests activation of β‐catenin signaling is contributing to hepatocyte proliferation, and deletion of β‐catenin leads to impaired hepatocyte proliferation and defective LR after CDE diet.

### LACK OF β‐CATENIN IN HEPATOCYTES IMPAIRS HEPATOCYTE PROLIFERATION AND PROMOTES INJURY FROM THE CDE DIET

**Figure 2:**
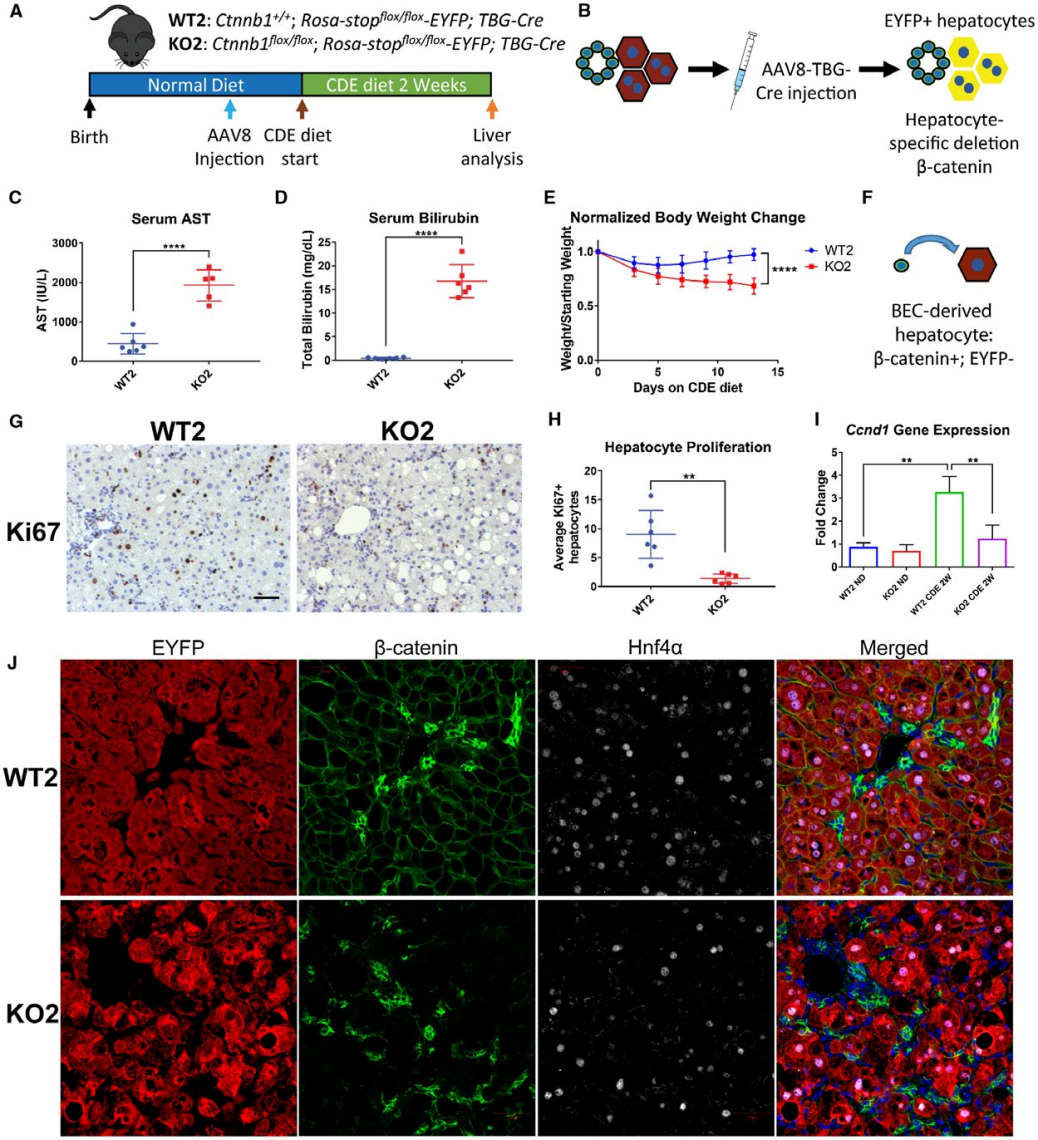
KO2 mice display severe liver injury and impaired hepatocyte proliferation after CDE diet–induced liver injury. (A) Male 24‐25‐day‐old *Ctnnb1^flox/flox^; Rosa‐stop^flox/flox^‐EYFP or Ctnnb1^+/+^; Rosa‐stop^flox/flox^‐EYFP *mice were injected with AAV8‐TBG‐Cre to generate KO2 and WT2 mice, respectively, followed by 12 days of wash‐out on normal diet before 2 weeks of CDE diet. (B) Schematic of cell labeling after AAV8‐TBG‐Cre injection, only hepatocytes (maroon hexagons) will be labeled with EYFP and will lose β‐catenin expression (black outline). BECs (green circles) will not be altered by AAV8‐TBG‐Cre injection. (C) Serum liver injury marker AST is significantly elevated in KO2 mice after CDE diet compared with WT2 mice (*t* test, **** = *P* < 0.0001). (D) Serum bilirubin is significantly elevated in KO2 mice after CDE diet compared with WT2 mice (*t* test, **** = *P* < 0.0001). (E) KO2 mice lose significantly more body weight compared with WT2 mice after CDE diet (WT2 n = 22, KO2 n =11, two‐way ANOVA, **** = *P* < 0.0001). (F) Schematic of fate tracing showing that any BECs that give rise to hepatocytes will be negative for EYFP and will express β‐catenin. (G) IHC shows many Ki67‐positive hepatocytes in WT2 mice but not in KO2 after CDE diet (scale bar 50 µm). (H) Quantification reveals fewer Ki67‐positive hepatocytes in KO2 mice after CDE diet (Mann‐Whitney U test, ** = *P *< 0.01). (I) Hepatic *Ccnd1* gene expression is significantly elevated in WT2 mice after CDE diet, but impaired in KO2 mice (one‐way ANOVA, ** = *P* < 0.01). (J) No BEC‐derived hepatocytes (EYFP negative, β‐catenin positive) were evident in either WT2 or KO2 mice after 2 weeks of CDE diet (scale bar 50 µm).

As KO1 mice on CDE diet displayed severe liver injury, impaired hepatocyte proliferation, and robust expansion of BECs, we hypothesized BEC differentiation to hepatocytes would be activated to mediate restoration of hepatocyte mass. To test this hypothesis, we generated KO2 mice.^23^ Genes in the *Rosa* locus are ubiquitously expressed, but prior to Cre recombination, a floxed stop codon inactivates expression of the *Rosa*‐driven EYFP reporter gene.^30^ AAV8 infects only hepatocytes, and greater than 99% of hepatocytes can be permanently labeled with EYFP after AAV8‐TBG‐Cre injection.^23^, ^25^, ^31^ We administered a single injection of AAV8‐TBG‐Cre and allowed 12‐day washout before administering CDE diet (Fig. 2A) to allow for clearance of residual viral genome.^32^ In KO2, hepatocytes lack β‐catenin expression and are permanently labeled with EYFP (Fig. 2B). Importantly, BECs are not infected with AAV8 and thus retain β‐catenin and do not express EYFP. Therefore, hepatocytes originating from BECs will be negative for EYFP and express β‐catenin (Fig. 2F). As controls, WT2 mice showed EYFP‐labeled hepatocytes that retain β‐catenin expression. IHC confirmed β‐catenin expression in hepatocytes in WT2 mice but not in KO2 mice (Supporting Fig. S2A).

KO2 on CDE diet, like KO1, showed severe liver injury reflected by significantly increased serum AST (Fig. 2C), serum ALT (Supporting Fig. S2D), and bilirubin levels (Fig. 2D) as compared with WT2. WT2 mice on CDE diet initially lost weight but began to recover after 5‐7 days. In contrast, KO2 mice continued to lose weight over the course of CDE diet administration (Fig. 2E), suggesting failure to recover from injury. Both WT2 and KO2 mice displayed expansion of BECs (Fig. S2A), confirmed by increased *Krt19* (Supporting Fig. S2F) and *Sox9* (Supporting Fig. S2G) expression. KO2 and WT2 mice developed fibrosis after 2 weeks of CDE diet, evident by increased Sirius Red staining (Supporting Fig. S2A) and increased expression of *Col1a1* (Supporting Fig. S2B) and *Acta2* (Supporting Fig. S2C). KO2 mice on CDE diet also displayed increased p21‐positive hepatocytes (Supporting Fig. S2A) and overall p21 protein levels (Supporting Fig. S2H).

When hepatocyte proliferation was assessed by Ki67 staining, WT2 mice also displayed robust periportal hepatocyte proliferation, which was nearly absent in KO2 mice (Fig. 2G,H). WT2 mice, like WT1, displayed increased *Ccnd1* gene (Fig. 2I) and protein expression (Supporting Fig. S2H) after CDE diet, which was significantly abrogated in KO2. This suggests severely impaired hepatocyte proliferation in KO2 mice after 2 weeks of CDE diet. We did not detect any β‐catenin‐positive, EYFP‐negative hepatocytes in KO2 (Fig. 2J), suggesting no BEC‐derived hepatocytes (Fig. 2F) were present at this time. Corroborating this observation, we did not detect an increase in total β‐catenin levels in KO2 mice after 2 weeks of CDE diet in comparison with KO2 mice left on normal diet. However, we did detect active β‐catenin in KO2 mice on CDE diet, which was absent in KO2 mice on normal diet (Supporting Fig. S2H). This suggests active‐β‐catenin is present in β‐catenin‐positive BEC compartment, and that BEC differentiation into hepatocytes had yet to occur.

### DEFECTIVE HEPATOCYTE PROLIFERATION IN KO2 MICE DRIVES BEC‐TO‐HEPATOCYTE DIFFERENTIATION AFTER CDE DIET‐INDUCED HEPATIC INJURY

**Figure 3:**
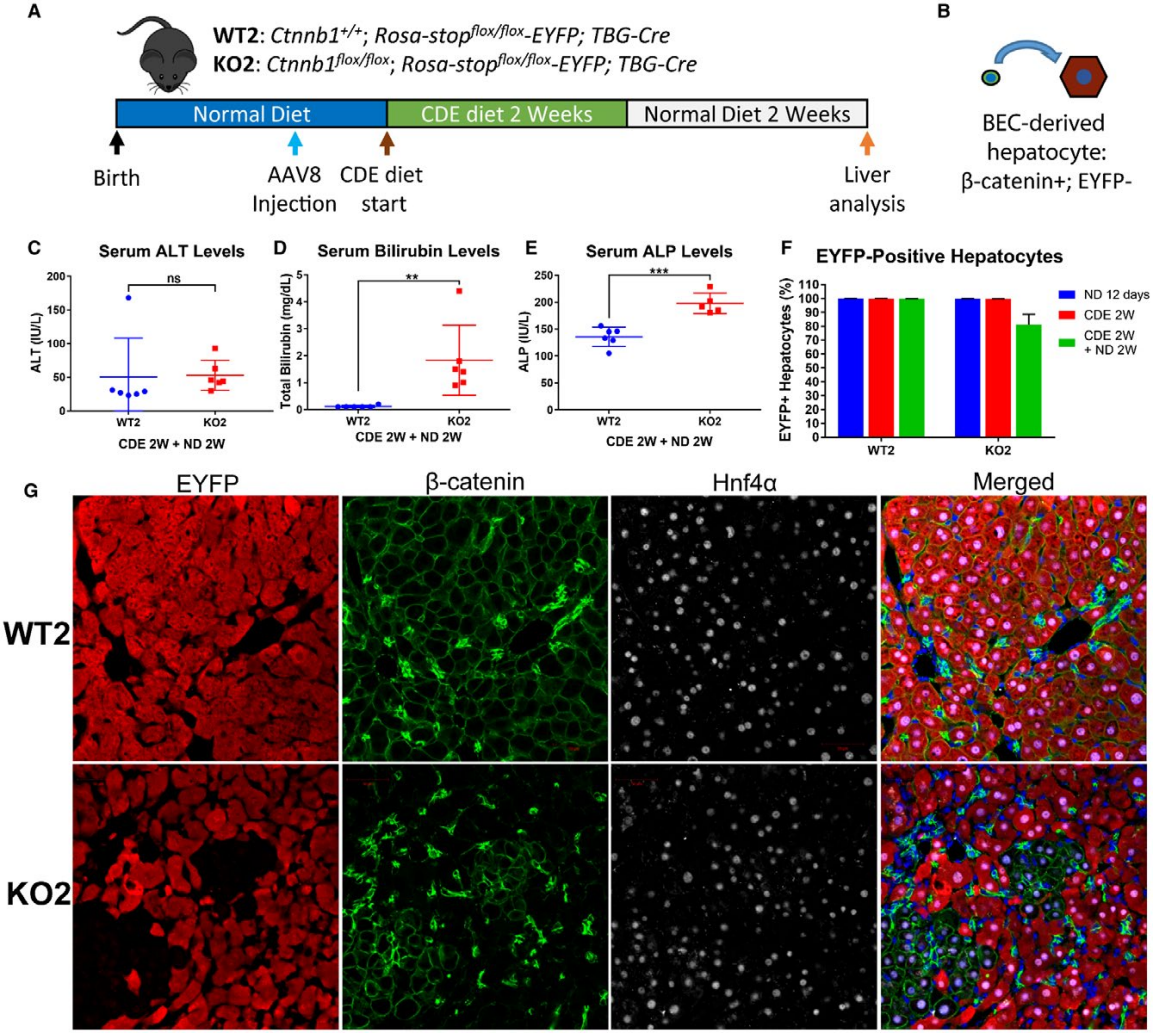
BEC‐derived hepatocytes appear in KO2 but not WT2 mice following recovery after CDE diet. (A) Male 24‐25‐day‐old *Ctnnb1^flox/flox^; Rosa‐stop^flox/flox^‐EYFP or Ctnnb1^+/+^; Rosa‐stop^flox/flox^‐EYFP *mice were injected with AAV8‐TBG‐Cre to generate KO2 and WT2 mice, respectively, followed by 12 days of wash‐out on normal diet before 2W of CDE diet followed by another 2W of recovery on normal chow. (B) Schematic of fate tracing depicting BECs that give rise to hepatocytes will be negative for EYFP and will express β‐catenin. (C) Serum ALT levels normalize after 2W of recovery post‐CDE diet in both WT2 and KO2 mice. (D) Serum bilirubin levels are reduced in KO2 mice compared with immediate post‐CDE‐diet injury levels but are still significantly elevated in comparison with WT2 mice following 2W of recovery (*t* test, ** = *P* < 0.01). (E) Serum ALP levels are significantly elevated in KO2 compared with WT2 mice after CDE 2W and 2W of recovery (*t* test, *** = *P* < 0.001). (F) Over 99% of hepatocytes are EYFP positive in WT2 and KO2 mice both prior to injury (ND 12 days) and after 2W of CDE diet–induced liver injury (CDE 2W). After recovery on normal diet following CDE diet, approximately 20% of periportal hepatocytes are EYFP negative in KO2 mice, whereas there is no reduction in EYFP‐positive hepatocytes in WT2 mice. (G) Hnf4α‐positive hepatocytes that are negative for EYFP and positive for β‐catenin appear in KO2 mice after CDE diet and recovery. No EYFP‐negative hepatocytes are apparent in WT2 mice (scale bar 50 µm). Abbreviations: ND, normal diet; W, weeks.

To facilitate BEC‐driven repair, we next administered CDE diet to WT2 and KO2 mice for 2 weeks, followed by recovery on normal diet for 2 weeks^17^, ^19^, ^33^ (Fig. 3A). Both WT2 and KO2 displayed normal serum ALT (Fig. 3C), although KO2 displayed slightly elevated serum bilirubin (Fig. 3D) and ALP (Fig. 3E) compared with WT2. Excitingly, in KO2 mice we detected clusters of β‐catenin‐positive, EYFP‐negative cells, which stained positively for hepatocyte marker Hnf4α (Fig. 3G), indicating BEC‐derived hepatocytes (Fig. 3B). We did not detect expansion of EYFP‐negative hepatocytes in WT2 mice, suggesting that BECs do not give rise to hepatocytes in animals when hepatocyte proliferation is not impaired, consistent with previous reports.^14^, ^23^, ^25^ An alternative explanation for these cells is that they were hepatocytes that escaped initial Cre recombination, which could result in EYFP‐negative, β‐catenin‐positive hepatocytes. However, we would predict the efficiency of AAV8‐TBG‐Cre to be the same in both WT2 and KO2 mice. Therefore, we quantified the number of EYFP‐positive/negative hepatocytes in our WT2 and KO2 mice over the course of CDE diet injury and recovery (Fig. 3F). First, we quantified the number of EYFP‐positive hepatocytes in WT2 and KO2 mice 12 days after injection with AAV8‐TBG‐Cre, corresponding to the initial labeling efficiency before the onset of liver injury. We found that greater than 99% of hepatocytes were EYFP positive, consistent with previous reports.^23^, ^25^ After 2 weeks of CDE diet, the percentage of EYFP‐negative hepatocytes was not significantly increased in either WT2 or KO2. However, after 2 weeks of recovery after CDE diet–induced liver injury, in KO2 mice approximately 20% of periportal hepatocytes were EYFP negative, whereas the percentage of EYFP‐negative hepatocytes in WT2 mice did not increase (Fig. 3F).

Correspondingly, tiled images (and serial higher‐magnification images from a representative area) from KO2 mice of representative lobes stained for β‐catenin revealed no clusters of β‐catenin‐positive hepatocytes after 2 weeks of CDE diet (Supporting Fig. S3A,B), whereas after 2 weeks of CDE diet followed by 2 weeks of recovery on normal diet, many clusters of β‐catenin‐positive hepatocytes were evident across the entire lobe (and serial higher‐magnification images from a representative area) (Supporting Fig. S3C,D). Likewise, hepatic *Ctnnb1* expression tended to increase in KO2 mice after CDE diet and recovery, although it was still significantly lower than WT2 mice. In age‐matched control mice left on normal diet for 6 weeks after AAV8‐TBG‐Cre injection, hepatic gene expression of *Ctnnb1* was greatly reduced in KO2 mice compared with WT2 mice (Supporting Fig. S3E), indicating lack of repopulation of β‐catenin‐positive hepatocytes in the absence of liver injury. These results, in combination with lack of expansion of EYFP‐negative hepatocytes in WT2 after CDE diet injury and recovery, support BEC‐to‐hepatocyte conversion in KO2 over expansion of Cre‐recombinase escaped cells.

### BEC‐DERIVED β‐CATENIN‐POSITIVE HEPATOCYTES ARE MORE PROLIFERATIVE THAN ENDOGENOUS HEPATOCYTES

**Figure 4:**
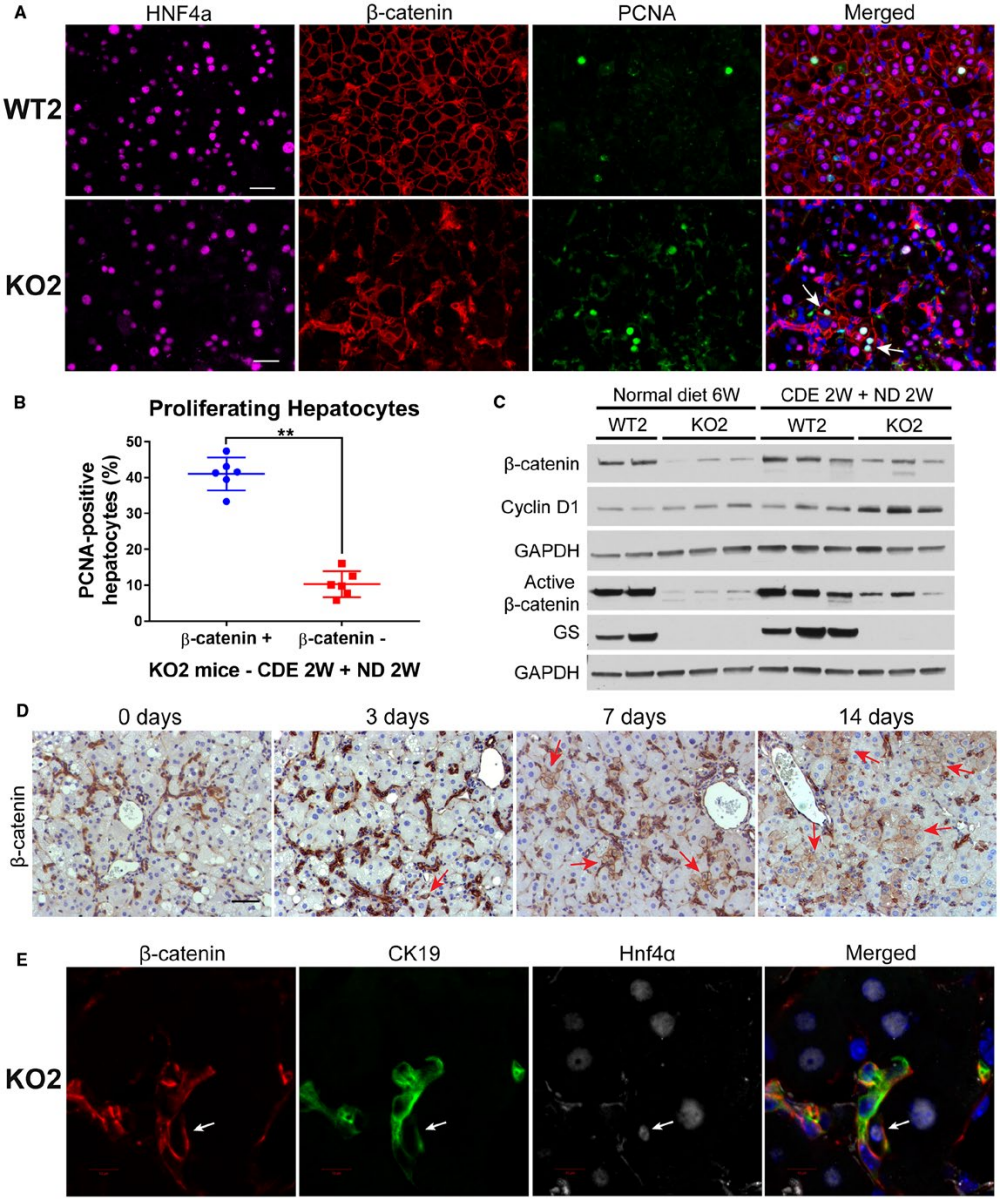
β‐catenin‐positive hepatocytes preferentially proliferate to restore lost hepatocyte mass. (A) The majority of BEC‐derived β‐catenin‐positive hepatocytes in a given cluster are PCNA positive (scale bar 50 µm). (B) Quantification of PCNA reveals β‐catenin‐positive hepatocytes are significantly more proliferative than surrounding β‐catenin‐negative hepatocytes (Mann‐Whitney U test, ** = *P* < 0.01). (C) There is an increase in total β‐catenin, active β‐catenin, and Cyclin D1 levels in KO2 mice after CDE diet and recovery compared with KO2 mice left on normal diet. Because all BEC‐derived hepatocytes are located in the periportal region, there is no re‐expression of pericentral β‐catenin target GS. (D) IHC staining for β‐catenin reveals no β‐catenin‐positive hepatocytes in KO2 mice after 2W on CDE diet with 0 days of recovery. However, a few β‐catenin‐positive hepatocytes are visible after 3 days of recovery (red arrow), with clusters of β‐catenin‐positive hepatocytes increasing in size from 7 to 14 days of recovery on normal diet (scale bar 50 µm). (E) β‐catenin‐positive cells that express both BEC marker CK19 and hepatocyte marker Hnf4α can be found in KO2 mice after 2W on CDE diet followed by 7 days of recovery on normal diet (scale bar 10 µm). Abbreviations: ND, normal diet; PCNA, proliferating cell nuclear antigen; W, weeks.

Analysis of hepatic sections from KO2 mice after 2 weeks of recovery on normal chow following 2 weeks of CDE diet showed that nearly all β‐catenin‐positive hepatocytes were located in clusters adjacent to BECs in the periportal region (Supporting Fig. S3B). This may also explain the lack of reappearance of pericentral β‐catenin target glutamine synthetase (GS)^26^, ^34^ in KO2 mice at this time (Fig. 4C). The reactive BEC response was extensive after 2 weeks of CDE diet and recovery, as evidenced by large numbers of epithelial cell adhesion molecule (EpCAM)–positive BECs (Supporting Fig. S4A). The extent of BEC expansion was similar in WT2 and KO2, as indicated by comparable hepatic expression of BEC markers *Krt19* (Supporting Fig. S4B) and *Sox9* (Supporting Fig. S4C). There was ongoing fibrosis in both WT2 and KO2 as evidenced by Sirius Red staining even after 2 weeks of recovery on normal diet (Supporting Fig. S4A), which is likely due to ongoing reactive BEC response, which is known to secrete profibrogenic cytokines.^35^ This level of fibrosis also indicated that repair of CDE diet–induced liver injury was not complete after 2 weeks of recovery on normal diet.

To determine if BEC‐derived, β‐catenin‐positive hepatocytes were driving restoration of hepatocyte mass in KO2 mice after CDE diet–induced liver injury, we performed triple immunofluorescence for Hnf4α, β‐catenin, and proliferating cell nuclear antigen (PCNA) (Fig. 4A). Within a given cluster of β‐catenin‐positive hepatocytes in KO2, the majority of β‐catenin‐positive hepatocytes stained positively for PCNA. Indeed, periportal β‐catenin‐positive hepatocytes were significantly more proliferative than surrounding β‐catenin‐negative hepatocytes in KO2 (Fig. 4B). Likewise, clusters of periportal hepatocytes in KO2 were strongly Cyclin D1 positive in comparison with surrounding hepatocytes (Supporting Fig. S4A, red arrows). KO2 also displayed increased total Cyclin D1 protein compared with WT2 after 2 weeks of CDE diet and recovery (Fig. 4C). There was also a noticeable increase in total and active‐β‐catenin in KO2 after recovery in comparison with age‐matched KO2 left on normal diet for 6 weeks (Fig. 4C), indicating repopulation by β‐catenin‐positive cells in KO2 during repair. Collectively, these results demonstrate BEC‐derived β‐catenin‐positive hepatocytes are preferentially proliferating to replenish lost hepatic mass in KO2 mice.

### BEC DIFFERENTIATION TO HEPATOCYTES OCCURS EARLY IN RECOVERY ON NORMAL DIET

To determine if the BEC‐derived hepatocytes after 2 weeks of recovery on normal diet express BEC markers, we performed triple immunofluorescence with a wide‐spectrum cytokeratin antibody (PanCK) in addition to EYFP and hepatocyte marker Hnf4α (Supporting Fig. S4D). EYFP‐negative hepatocytes were PanCK negative (Supporting Fig. S4D, white arrowhead), and as expected the majority of cytokeratin‐positive BECs were negative for EYFP. Interestingly, rare EYFP‐positive BECs were observed in both WT2 and KO2 mice (Supporting Fig. S4D, white arrows), potentially indicating hepatocyte‐to‐BEC transdifferentiation after CDE diet–induced liver injury. When we performed staining for a second BEC marker, EpCAM, we observed rare cells with hepatocyte‐like morphology, which were EpCAM positive in KO2 mice (Supporting Fig. S4A, black arrows). This finding correlates with the observations of EpCAM‐positive hepatocytes in human patients with BEC expansion after severe liver injury.^16^ These results demonstrate that after 2 weeks of recovery on normal diet, the majority of BEC‐derived hepatocytes in KO2 mice do not express BEC markers.

To further explore the timing of BEC‐to‐hepatocyte differentiation during recovery from CDE diet, we harvested KO2 mice after 3 days or 7 days of recovery on normal diet post‐CDE diet (Supporting Fig. S5A). Excitingly, very few small β‐catenin‐positive hepatocyte‐like cells are evident after 3 days of recovery, which grow into small clusters of β‐catenin‐positive cells with hepatocyte morphology after 7 days of recovery (Fig. 4D, red arrows). These clusters are even larger following 2 weeks of recovery on normal diet, suggesting potential clonal expansion of BEC‐derived, β‐catenin‐positive hepatocytes. Interestingly, serum ALT levels are dramatically reduced following 3 days of recovery on normal diet (Supporting Fig. S5B), whereas both serum bilirubin and ALP levels remain elevated after 3 days of recovery and only begin to normalize after 7 or 14 days of recovery, respectively (Supporting Fig. S5C,D), potentially suggesting abatement of acute liver injury may be required for expansion of these cells. Similar serum liver injury profiles and appearance of small β‐catenin‐positive hepatocytes were also observed in female KO2 mice after CDE diet and 3 days of recovery (data not shown), suggesting BEC‐to‐hepatocyte differentiation also occurs in female mice following recovery from CDE diet–induced liver injury.

We next investigated the expression of Hnf4α in these putative transdifferentiating BECs over time. After 2 weeks of CDE diet, virtually all β‐catenin‐positive cells were positive for CK19 and negative for Hnf4α (Supporting Fig. S5E). However, as early as after 3 days of recovery, we detected β‐catenin‐positive cells that were CK19 positive and weakly positive for Hnf4α (Supporting Fig. S5F). After 7 days of recovery on normal diet, rare β‐catenin‐positive cells were observed, which were strongly positive for both CK19 and Hnf4α (Fig. 4E). At this time point, clusters containing β‐catenin+/Hnf4α+/CK19− hepatocytes were evident (Supporting Fig. S5G). The rarity of the β‐catenin+/Hnf4α+/CK19+ cells in combination with the increased proliferation of β‐catenin‐positive hepatocytes suggests that few BECs differentiate into hepatocytes, which subsequently proliferate to restore lost hepatocyte mass in KO2 mice.

### BEC‐DERIVED HEPATOCYTES REPOPULATE THE LIVER DURING LONG‐TERM RECOVERY

**Figure 5:**
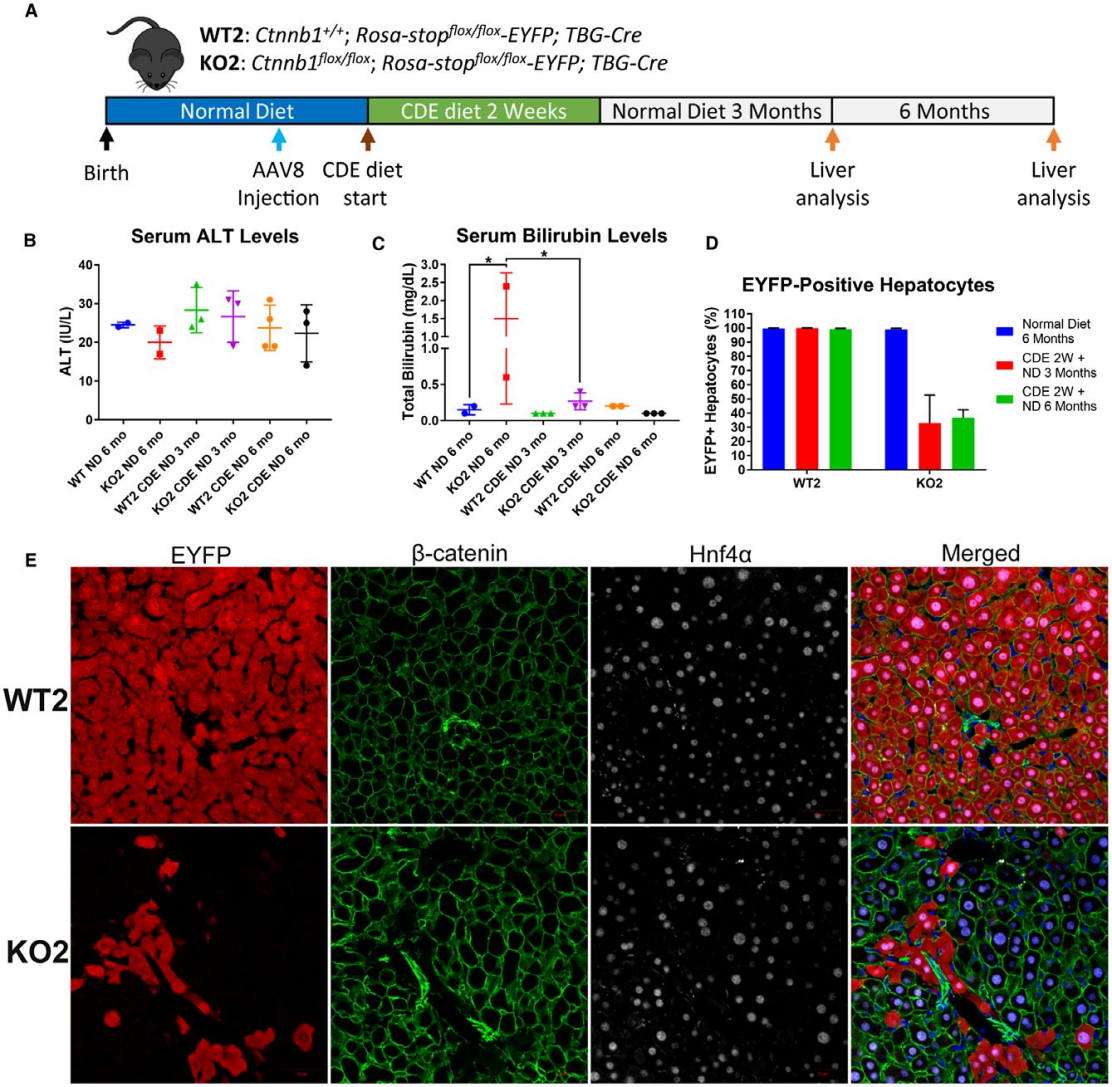
β‐catenin‐positive hepatocytes repopulate the majority of the liver during long‐term recovery. (A) After CDE diet treatment, WT2 and KO2 mice were allowed to recover on normal diet for either 3 or 6 months. As a control, WT2 and KO2 mice that were never exposed to CDE diet were traced for 6 months after AAV8 injection. (B) Serum ALT levels are normal in both WT2 and KO2 mice after long‐term recovery on normal diet. (C) Serum bilirubin levels are normal in both WT2 and KO2 mice on long‐term recovery on normal diet, although serum bilirubin is slightly elevated in control KO2 mice kept on normal diet for 6 months (one‐way ANOVA, * = *P *< 0.05). (D) There is no significant expansion of EYFP‐negative hepatocytes in control WT2 and KO2 mice left on normal diet for 6 months (blue bars), or in WT2 mice exposed to CDE diet for 2W and allowed to recover on normal diet for up to 6 months. However, in KO2 mice, up to 70% of hepatocytes are EYFP negative after 6 months of recovery on normal diet following CDE diet–induced liver injury. (E) Expansion of EYFP‐negative hepatocytes is only evident in KO2, not WT2, mice after 6 months of recovery on normal diet following CDE diet–induced liver injury. Abbreviations: mo, months; ND, normal diet; W, weeks.

Previous studies have demonstrated that transdifferentiated cells, such as hepatocyte‐derived BECs, may revert back to their original cell type when liver injury has abated.^14^ Alternatively, it has been shown that with persistent need for transdifferentiated cells, such as in mice that lack the intrahepatic biliary system, hepatocyte‐to‐BEC conversion is permanent and stable for life.^13^ To determine if BEC‐derived hepatocytes would persist and further repopulate the liver, KO2 mice were allowed to recover on normal diet for either 3 or 6 months after CDE diet (Fig. 5A). As a control, we traced WT2 and KO2 mice for 6 months on normal diet to determine the persistence of EYFP labeling in the absence of injury. Serum ALT levels were normal in both WT2 and KO2 (Fig. 5B,C). Interestingly, KO2 mice left on normal diet for 6 months showed minor elevation of bilirubin compared with WT2 (Fig. 5C). KO2 mice after long‐term recovery from CDE diet showed extensive expansion of EYFP‐negative, β‐catenin‐positive hepatocytes (Fig. 5E), as evidenced by central veins that were partially or completely GS positive (Supporting Fig. S6A). When we quantified the number of EYFP‐positive hepatocytes, we found that in control WT2 and KO2 left on normal diet for 6 months, there was no significant expansion of EYFP‐negative hepatocytes, similar to WT2 after long‐term recovery from CDE diet (Fig. 5D). However, in KO2 after 3 months of recovery from CDE diet, up to 70% of hepatocytes were EYFP negative, and this number remained stable after 6 months of recovery. In control KO2 mice left on normal diet for 6 months, virtually all EYFP‐positive hepatocytes were β‐catenin negative (Supporting Fig. S6B), as confirmed by β‐catenin IHC (Supporting Fig. S6C). Hepatic expression of *Ctnnb1* was significantly reduced in KO2 mice left on normal diet for 6 months compared with control WT2 mice, whereas expression of *Ctnnb1* in KO2 after recovery from CDE diet began to approach WT2 levels (Supporting Fig. S6D). The lack of expansion of EYFP‐negative hepatocytes in WT2 mice even after 6 months of recovery post‐CDE diet, combined with the lack of expansion of EYFP‐negative hepatocytes in KO2 left on normal diet, further supports our hypothesis that BEC‐derived hepatocytes repopulate the liver in KO2 after CDE diet–induced liver injury.

### *IN VIVO Ctnnb1* RNAi IMPAIRS HEPATOCYTE PROLIFERATION AFTER CDE DIET‐INDUCED LIVER INJURY

**Figure 6:**
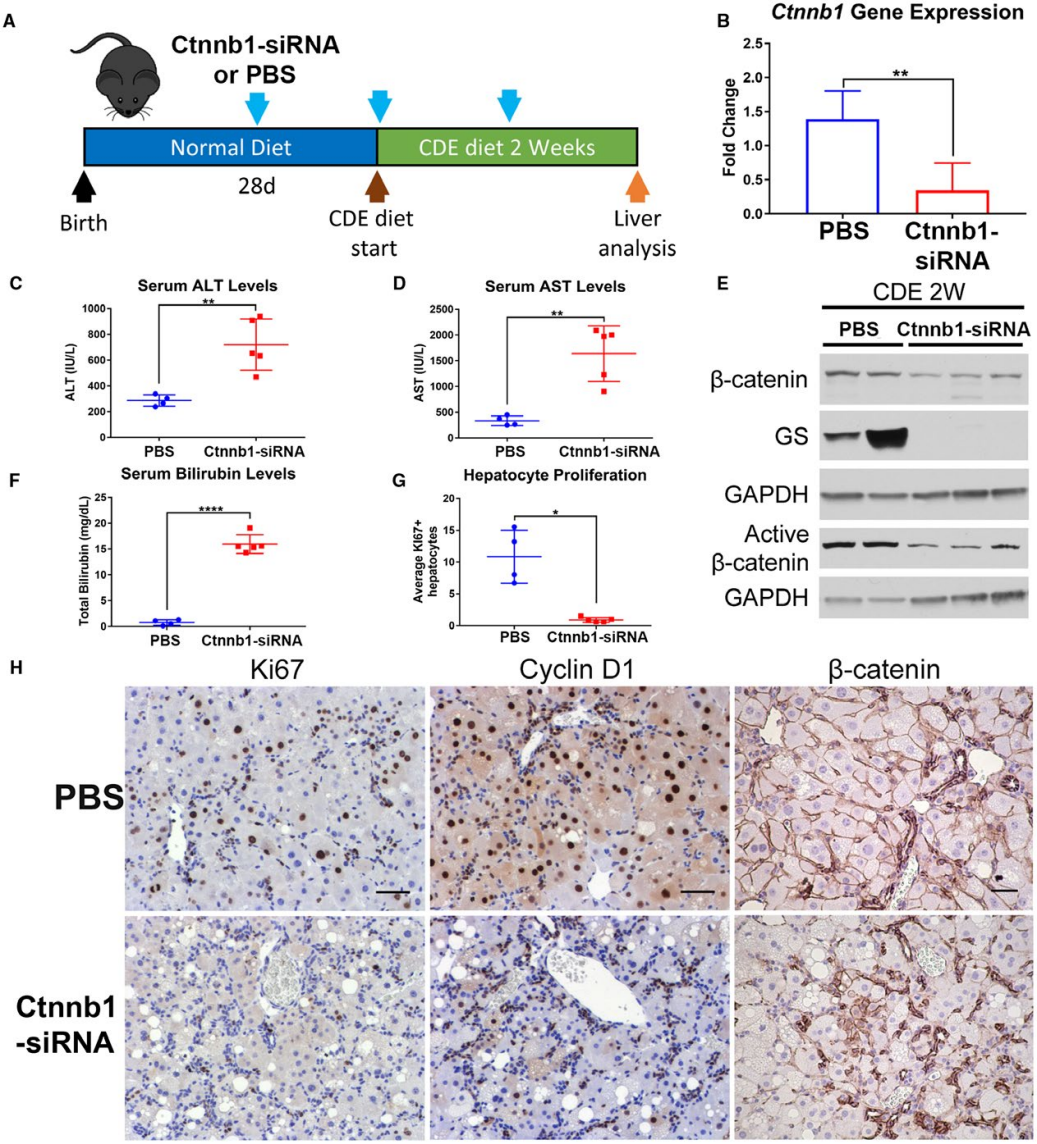
Hepatocyte‐specific *in vivo* *Ctnnb1* siRNA induces severe liver injury and a block of hepatocyte proliferation in CDE diet–fed mice. (A) C57BL6/NJ mice were placed on CDE diet for 2 weeks. Mice were given weekly subcutaneous injections of *Ctnnb1*‐siRNA dissolved in PBS or PBS control, 1 week prior to CDE diet administration and throughout the course of CDE diet feeding. (B) RT‐PCR analysis of *Ctnnb1* gene expression in whole liver lysates of *Ctnnb1*‐siRNA or PBS‐injected mice on CDE diet (*t* test, ** = *P* < 0.01). (C) Significantly elevated serum ALT levels in *Ctnnb1*‐siRNA‐injected mice compared with PBS controls (*t* test, ** = *P* < 0.01). (D) Significantly elevated serum AST levels in *Ctnnb1*‐siRNA‐injected mice compared with PBS controls (*t* test, ** = *P* < 0.01). (E) Whole liver lysate reveals reduction in total β‐catenin and active β‐catenin, as well as complete loss of β‐catenin‐target GS in *Ctnnb1*‐siRNA‐injected mice on CDE diet. (F) Significantly elevated serum bilirubin levels in *Ctnnb1*‐siRNA‐injected mice compared with PBS controls (*t* test, **** = *P *< 0.0001). (G) Quantification reveals significantly fewer Ki67‐positive hepatocytes in *Ctnnb1*‐siRNA‐injected mice compared with PBS‐injected controls (Mann‐Whitney U test, * = *P* < 0.05). (H) IHC staining reveals robust Ki67 staining of hepatocytes in PBS but not *Ctnnb1*‐siRNA‐injected animals. Cyclin D1 staining is significantly reduced in *Ctnnb1*‐siRNA‐injected mice, which also display a lack of hepatocyte‐specific β‐catenin staining (scale bar 50 µm). Abbreviation: d, day.

To prove BECs were indeed giving rise to hepatocytes in our model, we sought to perform direct lineage tracing of BECs using tamoxifen‐inducible *Krt19*‐*Cre* to label BECs with the reporter tdTomato. However, the use of Cre recombinase to label BECs precluded the use of Cre recombinase to delete *Ctnnb1* specifically in hepatocytes. To achieve labeling of BECs with a reporter and simultaneous knockdown of *Ctnnb1* expression in hepatocytes, we used *Ctnnb1*‐siRNA conjugated to hepatocyte‐targeting ligand N‐acetylgalactosamine.^36^ To validate that *Ctnnb1*‐siRNA injection would recapitulate the phenotype of CDE diet–fed mice with genetic hepatocyte‐specific *Ctnnb1* deletion, we performed weekly subcutaneous injections of *Ctnnb1*‐siRNA in mice fed CDE diet. The first injection was performed 1 week before CDE diet exposure to ensure reduced β‐catenin levels in hepatocytes at the time of CDE diet administration (Fig. 6A).

Real‐time PCR analysis demonstrated significant reduction of hepatic *Ctnnb1* gene expression in *Ctnnb1*‐siRNA‐injected mice compared with PBS‐injected control mice on CDE diet (Fig. 6B). *Ctnnb1*‐siRNA‐injected mice fed CDE diet for 2 weeks displayed significantly elevated serum ALT (Fig. 6C), AST (Fig. 6D), and bilirubin levels (Fig. 6F) compared with control PBS‐injected mice. We additionally confirmed reduction in both total and active β‐catenin protein levels, as well as loss of expression of β‐catenin downstream target GS, in *Ctnnb1*‐siRNA‐injected mice on CDE diet (Fig. 6E). Staining for Ki67 displayed robust hepatocyte proliferation in PBS‐injected controls but not *Ctnnb1*‐siRNA‐injected mice on CDE diet (Fig. 6H), which was confirmed by quantification (Fig. 6G). There was also a notable decrease in Cyclin D1 staining in the livers of *Ctnnb1*‐siRNA‐injected mice on CDE diet (Fig. 6H). Finally, IHC for β‐catenin confirmed loss of β‐catenin expression specifically in hepatocytes of *Ctnnb1*‐siRNA‐injected mice (Fig. 6H). Collectively, these results demonstrate that *Ctnnb1*‐siRNA‐injected mice display increased liver injury and impairment of hepatocyte proliferation.

### Becs Give Rise To Hepatocytes In *Ctnnb1* Rnai‐Treated Mice After CDE Diet‐Induced Liver Injury

**Figure 7:**
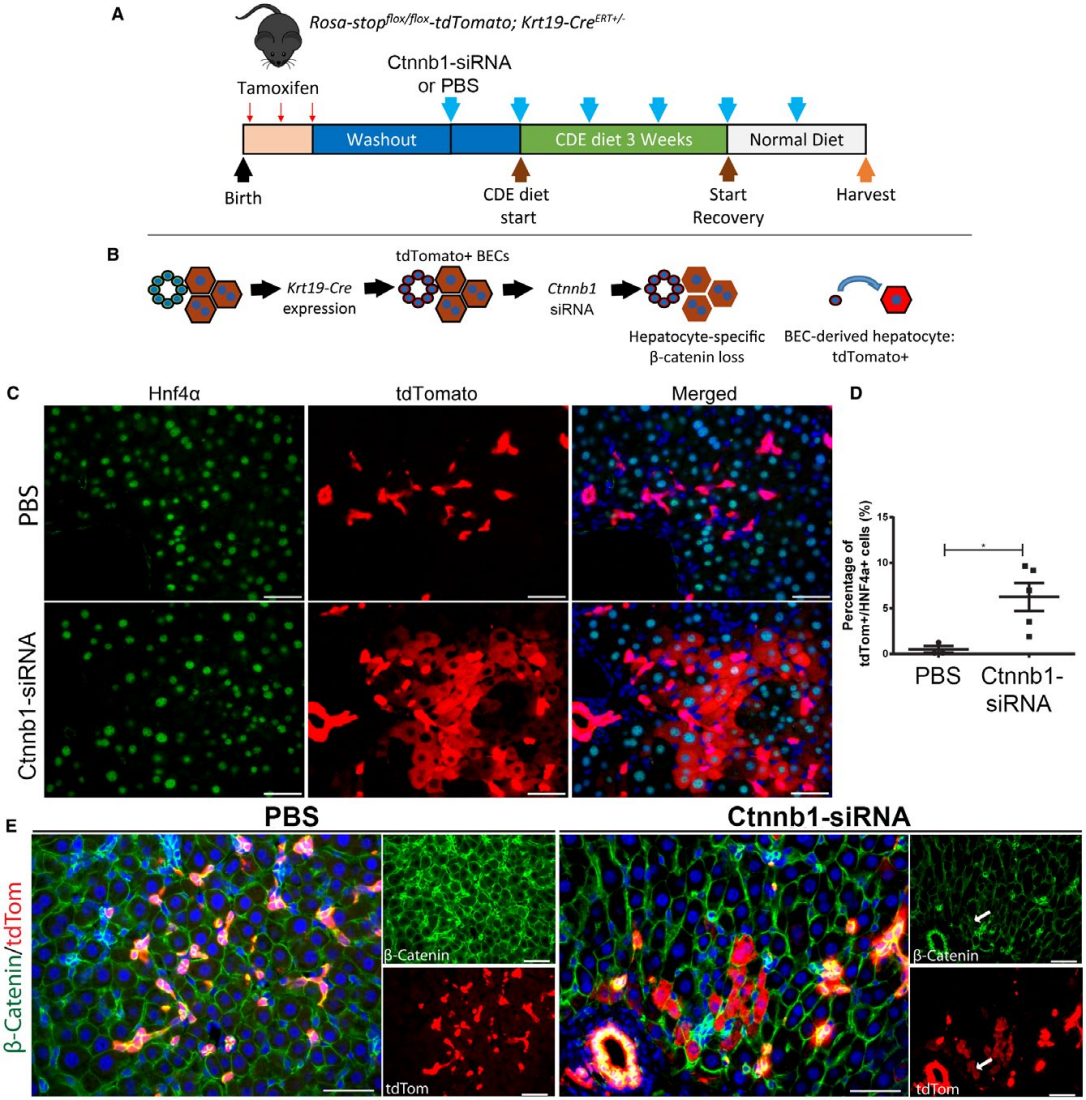
BEC‐derived hepatocytes appear in *Ctnnb1*‐siRNA but not PBS‐treated mice following recovery period after CDE diet. (A) *Krt19*‐Cre^ERT+/‐ ^mice were given tamoxifen during postnatal week 1, followed by a 2‐week washout. Mice were given subcutaneous injections of either *Ctnnb1*‐siRNA or PBS 1 week prior to CDE diet as control, and injected weekly throughout the course of the experiment. Mice remained on CDE diet for 3 weeks followed by a 2‐week recovery on normal diet. (B) After tamoxifen administration, BECs will be labeled with tdTomato, and *Ctnnb1*‐siRNA injections induce hepatocyte‐specific knockdown of *Ctnnb1* expression. Therefore, a BEC‐derived hepatocyte will be tdTomato positive and re‐express β‐catenin. (C) In control PBS‐injected mice after CDE diet and recovery, BECs but not hepatocytes are tdTomato positive. In *Ctnnb1*‐siRNA‐injected mice, clusters of tdTomato‐positive, Hnf4α‐positive hepatocytes are evident, demonstrating BEC‐to‐hepatocyte conversion (scale bar 100 µm). (D) Quantification of tdTomato, Hnf4α double‐positive cells reveals significantly more BEC‐derived hepatocytes in *Ctnnb1*‐siRNA‐injected mice compared with PBS‐injected controls (*t* test, * = *P* < 0.05). (E) In control PBS‐injected mice, both BECs and hepatocytes are β‐catenin positive, but only BECs are tdTomato positive. In *Ctnnb1*‐siRNA‐injected mice, hepatocytes lose cytoplasmic β‐catenin‐staining and clusters of tdTomato‐positive hepatocytes (white arrows) are located periportally (scale bar 100 µm).

**Figure 8:**
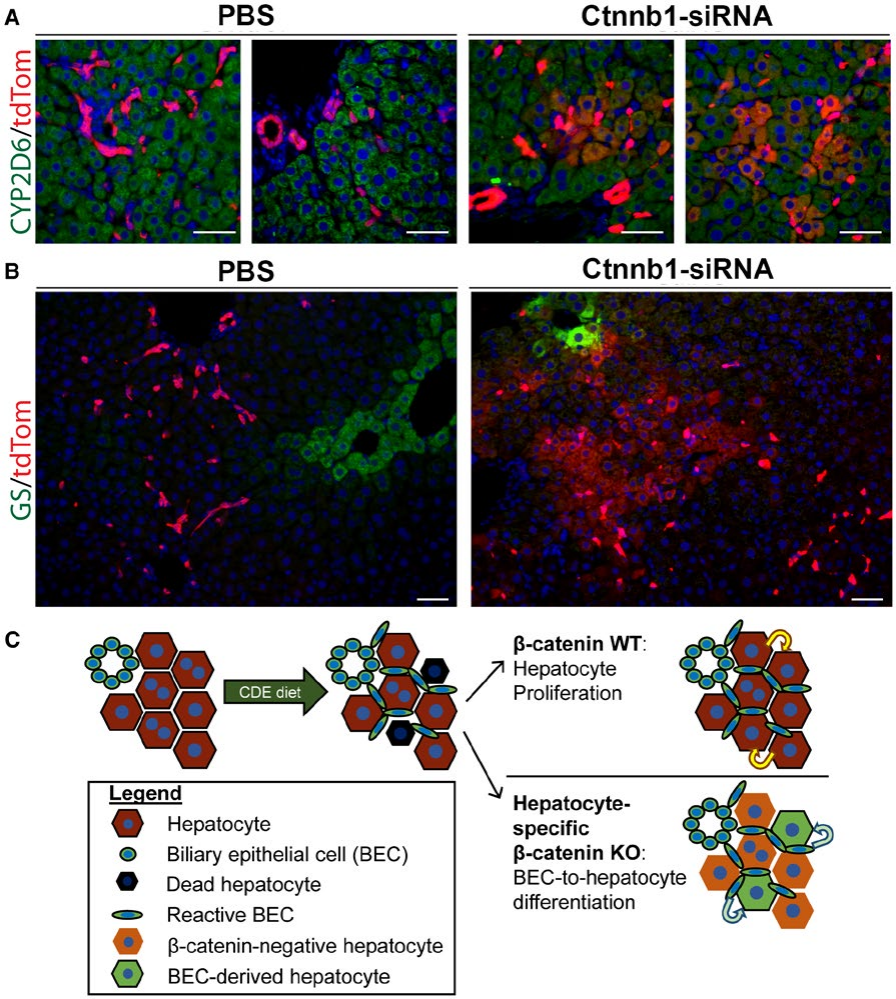
BEC‐derived hepatocytes express mature hepatocyte markers. (A) BEC‐derived tdTomato‐positive hepatocytes are positive for hepatocyte marker CYP2D6 in *Ctnnb1*‐siRNA‐injected mice (scale bar 100 µm). (B) BEC‐derived tdTomato‐positive hepatocytes in *Ctnnb1*‐siRNA‐injected mice extend all the way to the central vein, resulting in re‐expression of β‐catenin‐target GS (scale bar 100 µm). (C) Schematic illustrating key findings: When hepatocyte proliferation is intact (β‐catenin WT hepatocytes) after CDE diet–induced liver injury, liver regeneration is driven by hepatocyte proliferation. When mice with hepatocyte‐specific loss of β‐catenin are subjected to CDE diet, BEC‐to‐hepatocyte differentiation occurs to facilitate liver regeneration.

Having proved that injection of *Ctnnb1*‐siRNA was sufficient to block hepatocyte proliferation in mice on CDE diet, we developed a mouse model of *Krt19*‐Cre^ERT+/‐^‐driven BEC lineage tracing^17^ in combination with hepatocyte‐specific *Ctnnb1* knockdown. To label BECs, tamoxifen was injected in *Krt19*‐Cre^ERT+/‐ ^mice during postnatal week 1, followed by a 2‐week washout period to ensure elimination of any residual tamoxifen (Fig. 7A). Next, these mice were given weekly injections of *Ctnnb1*‐siRNA throughout the course of CDE diet and recovery (Fig. 7A). In this model, BEC‐derived hepatocytes can be directly traced because they will be tdTomato‐positive (Fig. 7B). Prior to CDE diet administration, we verified that early postnatal tamoxifen administration labeled only BECs and not surrounding hepatocytes (Supporting Fig. S7A), implying that any tdTomato‐positive hepatocytes would have to arise from a pre‐existing tdTomato‐positive BEC. We additionally determined the *Krt19*‐Cre^ERT+/‐ ^recombination efficiency in BECs in this model to be approximately 50% (Supporting Fig. S7B). After CDE diet and recovery, serum liver injury markers were approaching normal levels in both PBS and *Ctnnb1*‐siRNA‐injected mice (Supporting Fig. S7B‐D). Excitingly, clusters of tdTomato‐positive hepatocytes that were also Hnf4α positive (Fig. 7C) and CYP2D6 positive (Fig. 8A) were evident in the *Ctnnb1*‐siRNA‐injected mice but not in the mice injected with PBS as a control. Quantification revealed that approximately 6% of hepatocytes were tdTomato positive in *Ctnnb1*‐siRNA‐injected mice after CDE diet and recovery (Fig. 7D).

Immunofluorescence of β‐catenin was strongly detected in the hepatocytes of PBS‐injected mice but was significantly reduced in *Ctnnb1*‐siRNA‐injected mice (Fig. 7E). Importantly, tdTomato‐positive hepatocytes were located adjacent to clusters of β‐catenin‐positive BECs in *Ctnnb1*‐siRNA‐injected mice. Finally, clusters of tdTomato‐positive hepatocytes in *Ctnnb1*‐siRNA‐injected mice would occasionally extend all the way to the central vein, resulting in localized re‐expression of β‐catenin target GS (Fig. 8B). All together, these results demonstrate that BECs give rise to hepatocytes when hepatocyte proliferation is impaired, which was achieved in our model through the loss of β‐catenin expression in hepatocytes in mice exposed to CDE diet–induced liver injury (Fig. 8C).

## Discussion

The role of BECs in mediating LR has remained controversial, as several studies have reported a limited role for BEC differentiation to hepatocytes.^23^, ^24^, ^25^ Recent studies have demonstrated that widespread hepatocyte senescence,^17^ hepatocyte ablation,^20^ impaired hepatocyte proliferation,^19^ or prolonged exposure to severe liver injury^37^ can trigger differentiation of BECs to hepatocytes. Interestingly, a previous study reported ductular reaction and BEC‐to‐hepatocyte differentiation in aged *Albumin*‐Cre β‐catenin KO livers,^38^ suggesting expression of β‐catenin in hepatocytes is important for long‐term liver health. Here, we demonstrate that hepatocyte‐specific loss of β‐catenin expression in combination with CDE diet–induced liver injury triggers severe impairment of hepatocyte proliferation and leads to unequivocal repopulation of liver with BEC‐derived hepatocytes.

Genetic loss of β‐catenin in hepatocytes led to extensive injury in the CDE diet model with robust expansion of BECs in both male and female mice (data not shown). Further, knockdown of *Ctnnb1* expression in hepatocytes via siRNA also impaired hepatocyte proliferation after CDE diet. Intriguingly, BEC expansion occurred normally despite β‐catenin loss in these cells in *Albumin‐Cre* *Ctnnb1* KO mice on CDE diet. However, the appearance of active‐β‐catenin in KO2 mice, which retained β‐catenin expression in BECs, suggests activation of β‐catenin signaling in this compartment during CDE diet. This observation is in accordance with previous findings that activation of β‐catenin in reactive BECs promotes differentiation toward the hepatocyte lineage.^39^ In our model, appearance of BEC‐derived hepatocytes was evident as early as 3 days of recovery on basal diet after 2 weeks of CDE diet, when cells positive for both BEC and hepatocyte markers could be observed. Excitingly, Hnf4α+/CK19+ cells have also been observed in a recent study demonstrating BEC‐derived hepatocytes during long‐term severe liver injury.^37^ Our results suggest that differentiation of BECs to hepatocytes is limited or impaired during ongoing injury and that a period of recovery is necessary to allow BEC differentiation to hepatocytes, as also shown elsewhere.^17^, ^19^, ^33^

Greater than 99% of hepatocytes were labeled with EYFP after AAV8‐TBG‐Cre injection, and after 6 months of normal diet, the numbers of EYFP‐negative hepatocytes in both WT2 and KO2 mice were not significantly increased. Additionally, over 99% of hepatocytes were still EYFP positive in both WT2 and KO2 mice after 2 weeks of CDE diet, suggesting no significant population of hepatocytes escaping initial Cre‐recombination, as these cells would express β‐catenin and would be expected to have a proliferative advantage during CDE diet–induced liver injury. In this negative tracing model, we found that after recovery, approximately 20% of periportal hepatocytes were BEC derived. Interestingly, in our positive lineage tracing model, we found only 6% of hepatocytes were BEC derived. This is attributable to the fact that initially only around 50% of BECs were labeled with tdTomato in this model, suggesting potentially 12% of hepatocytes were BEC derived in our model after factoring in the limited recombination efficiency. Furthermore, newly BEC‐derived hepatocytes may be susceptible to siRNA‐mediated *Ctnnb1* knockdown, potentially reducing their proliferation during recovery from CDE diet, further reducing tdTomato‐positive hepatocyte numbers.

Although our data convincingly prove the differentiation and long‐term survival of BEC‐derived hepatocytes, much remains to be elucidated about both the long‐term effects of BEC‐derived hepatocytes on liver health and the mechanisms underlying BEC‐to‐hepatocyte differentiation. For instance, it will be interesting to determine if BEC‐derived hepatocytes are equally as adept at promoting resolution of liver injury, such as resolution of fibrosis. Additionally, although factors that promote ductular reaction are better known,^40^ our knowledge of the factors promoting BEC differentiation to hepatocytes is much more limited. Because our model establishes a clear timeline for BEC differentiation to hepatocytes, it will be invaluable in determining the factors that underlie this process. For example, as we have demonstrated the importance of β‐catenin signaling in LR, this would potentially allow promotion of BEC‐to‐hepatocyte differentiation while circumventing activation of oncogenic β‐catenin signaling in patients. Additionally, it will be important to determine the threshold of hepatic impairment that triggers BEC‐to‐hepatocyte differentiation, as this may lead to the development of biomarkers for patients with severe liver disease that are amenable to such treatments. These would be important steps in developing regenerative medicine therapies for the treatment of chronic liver disease.

## Potential conflict of interest

Dr. Abrams owns stock in Dicerna Pharmaceuticals.

## References

[1] Kochanek KD , Murphy SL , Xu J , Tejada‐Vera B . Deaths: final data for 2014. Natl Vital Stat Rep 2016;65:1‐122. 27378572

[2] Kim WR , Lake JR , Smith JM , Skeans MA , Schladt DP , Edwards EB , et al. OPTN/SRTR 2015 Annual Data Report: Liver. Am J Transplant 2017;17(Suppl. 1):174‐251. 28052604 10.1111/ajt.14126

[3] Carpino G , Renzi A , Onori P , Gaudio E . Role of hepatic progenitor cells in nonalcoholic fatty liver disease development: cellular cross‐talks and molecular networks. Int J Mol Sci 2013;14:20112‐20130. 24113587 10.3390/ijms141020112 PMC3821605

[4] **Zhang** L , **Theise** N , Chua M , Reid LM . The stem cell niche of human livers: symmetry between development and regeneration. Hepatology 2008;48:1598‐1607. 18972441 10.1002/hep.22516

[5] Gouw AS , Clouston AD , Theise ND . Ductular reactions in human liver: diversity at the interface. Hepatology 2011;54:1853‐1863. 21983984 10.1002/hep.24613

[6] Lowes KN , Brennan BA , Yeoh GC , Olynyk JK . Oval cell numbers in human chronic liver diseases are directly related to disease severity. Am J Pathol 1999;154:537‐541. 10027411 10.1016/S0002-9440(10)65299-6 PMC1849988

[7] Libbrecht L , Roskams T . Hepatic progenitor cells in human liver diseases. Semin Cell Dev Biol 2002;13:389‐396. 12468238 10.1016/s1084952102001258

[8] Carpentier R , Suñer RE , van Hul N , Kopp JL , Beaudry JB , Cordi S , et al. Embryonic ductal plate cells give rise to cholangiocytes, periportal hepatocytes, and adult liver progenitor cells. Gastroenterology 2011;141:1432‐1438.e1431‐1434. 21708104 10.1053/j.gastro.2011.06.049 PMC3494970

[9] **Huch** M , **Dorrell** C , Boj SF , van Es JH , Li VS , van de Wetering M , et al. *In vitro* expansion of single Lgr5+ liver stem cells induced by Wnt‐driven regeneration. Nature 2013;494:247‐250. 23354049 10.1038/nature11826 PMC3634804

[10] Duncan AW , Dorrell C , Grompe M . Stem cells and liver regeneration. Gastroenterology 2009;137:466‐481. 19470389 10.1053/j.gastro.2009.05.044 PMC3136245

[11] Thorgeirsson SS . Hepatic stem cells in liver regeneration. FASEB J 1996;10:1249‐1256. 8836038

[12] Lin S , Nascimento EM , Gajera CR , Chen L , Neuhöfer P , Garbuzov A , et al. Distributed hepatocytes expressing telomerase repopulate the liver in homeostasis and injury. Nature 2018;556:244‐248. 29618815 10.1038/s41586-018-0004-7 PMC5895494

[13] Schaub JR , Huppert KA , Kurial SNT , Hsu BY , Cast AE , Donnelly B , et al. De novo formation of the biliary system by TGFβ‐mediated hepatocyte transdifferentiation. Nature 2018;557:247‐251. 29720662 10.1038/s41586-018-0075-5 PMC6597492

[14] Tarlow BD , Pelz C , Naugler WE , Wakefield L , Wilson EM , Finegold MJ , et al. Bipotential adult liver progenitors are derived from chronically injured mature hepatocytes. Cell Stem Cell 2014;15:605‐618. 25312494 10.1016/j.stem.2014.09.008 PMC4254170

[15] Stueck AE , Wanless IR . Hepatocyte buds derived from progenitor cells repopulate regions of parenchymal extinction in human cirrhosis. Hepatology 2015;61:1696‐1707. 25644399 10.1002/hep.27706

[16] Michalopoulos GK , Khan Z . Liver stem cells: experimental findings and implications for human liver disease. Gastroenterology 2015;149:876‐882. 26278502 10.1053/j.gastro.2015.08.004 PMC4584191

[17] Lu WY , Bird TG , Boulter L , Tsuchiya A , Cole AM , Hay T , et al. Hepatic progenitor cells of biliary origin with liver repopulation capacity. Nat Cell Biol 2015;17:971‐983. 26192438 10.1038/ncb3203 PMC4612439

[18] Alison M , Golding M , Lalani EN , Nagy P , Thorgeirsson S , Sarraf C . Wholesale hepatocytic differentiation in the rat from ductular oval cells, the progeny of biliary stem cells. J Hepatol 1997;26:343‐352. 9059956 10.1016/s0168-8278(97)80051-7

[19] **Raven** A , **Lu** WY , Man TY , Ferreira‐Gonzalez S , O'Duibhir E , Dwyer BJ , et al. Cholangiocytes act as facultative liver stem cells during impaired hepatocyte regeneration. Nature 2017;547:350‐354. 28700576 10.1038/nature23015 PMC5522613

[20] Choi TY , Ninov N , Stainier DY , Shin D . Extensive conversion of hepatic biliary epithelial cells to hepatocytes after near total loss of hepatocytes in zebrafish. Gastroenterology 2014;146:776‐788. 24148620 10.1053/j.gastro.2013.10.019 PMC3943869

[21] Shinozuka H , Lombardi B , Sell S , Iammarino RM . Early histological and functional alterations of ethionine liver carcinogenesis in rats fed a choline‐deficient diet. Cancer Res 1978;38:1092‐1098. 76508

[22] Akhurst B , Croager EJ , Farley‐Roche CA , Ong JK , Dumble ML , Knight B , et al. A modified choline‐deficient, ethionine‐supplemented diet protocol effectively induces oval cells in mouse liver. Hepatology 2001;34:519‐522. 11526537 10.1053/jhep.2001.26751

[23] **Yanger** K , **Knigin** D , Zong Y , Maggs L , Gu G , Akiyama H , et al. Adult hepatocytes are generated by self‐duplication rather than stem cell differentiation. Cell Stem Cell 2014;15:340‐349. 25130492 10.1016/j.stem.2014.06.003 PMC4505916

[24] Tarlow BD , Finegold MJ , Grompe M . Clonal tracing of Sox9+ liver progenitors in mouse oval cell injury. Hepatology 2014;60:278‐289. 24700457 10.1002/hep.27084 PMC4077948

[25] Schaub JR , Malato Y , Gormond C , Willenbring H . Evidence against a stem cell origin of new hepatocytes in a common mouse model of chronic liver injury. Cell Rep 2014;8:933‐939. 25131204 10.1016/j.celrep.2014.07.003 PMC4376310

[26] Yang J , Mowry LE , Nejak‐Bowen KN , Okabe H , Diegel CR , Lang RA , et al. β‐catenin signaling in murine liver zonation and regeneration: a Wnt‐Wnt situation!. Hepatology 2014;60:964‐976. 24700412 10.1002/hep.27082 PMC4139486

[27] Monga SP . β‐catenin signaling and roles in liver homeostasis, injury, and tumorigenesis. Gastroenterology 2015;148:1294‐1310. 25747274 10.1053/j.gastro.2015.02.056 PMC4494085

[28] Tan X , Behari J , Cieply B , Michalopoulos GK , Monga SP . Conditional deletion of beta‐catenin reveals its role in liver growth and regeneration. Gastroenterology 2006;131:1561‐1572. 17101329 10.1053/j.gastro.2006.08.042

[29] Lai C , Pursell N , Gierut J , Saxena U , Zhou W , Dills M , et al. Specific inhibition of hepatic lactate dehydrogenase reduces oxalate production in mouse models of primary hyperoxaluria. Mol Ther 2018;26:1983‐1995. 29914758 10.1016/j.ymthe.2018.05.016 PMC6094358

[30] Soriano P . Generalized lacZ expression with the ROSA26 Cre reporter strain. Nat Genet 1999;21:70‐71. 9916792 10.1038/5007

[31] Malato Y , Naqvi S , Schürmann N , Ng R , Wang B , Zape J , et al. Fate tracing of mature hepatocytes in mouse liver homeostasis and regeneration. J Clin Invest 2011;121:4850‐4860. 22105172 10.1172/JCI59261 PMC3226005

[32] Zincarelli C , Soltys S , Rengo G , Rabinowitz JE . Analysis of AAV serotypes 1–9 mediated gene expression and tropism in mice after systemic injection. Mol Ther 2008;16:1073‐1080. 18414476 10.1038/mt.2008.76

[33] Shin S , Upadhyay N , Greenbaum LE , Kaestner KH . Ablation of Foxl1‐Cre‐labeled hepatic progenitor cells and their descendants impairs recovery of mice from liver injury. Gastroenterology 2015;148:192 ‐ 202.e193. 25286440 10.1053/j.gastro.2014.09.039 PMC4387775

[34] Cadoret A , Ovejero C , Terris B , Souil E , Lévy L , Lamers WH , et al. New targets of beta‐catenin signaling in the liver are involved in the glutamine metabolism. Oncogene 2002;21:8293‐8301. 12447692 10.1038/sj.onc.1206118

[35] Svegliati‐Baroni G , De Minicis S , Marzioni M . Hepatic fibrogenesis in response to chronic liver injury: novel insights on the role of cell‐to‐cell interaction and transition. Liver Int 2008;28:1052‐1064. 18783548 10.1111/j.1478-3231.2008.01825.x

[36] Nair JK , Willoughby JL , Chan A , Charisse K , Alam MR , Wang Q , et al. Multivalent N‐acetylgalactosamine‐conjugated siRNA localizes in hepatocytes and elicits robust RNAi‐mediated gene silencing. J Am Chem Soc 2014;136:16958‐16961. 25434769 10.1021/ja505986a

[37] **Deng** X , **Zhang** X , **Li** W , Feng RX , Li L , Yi GR , et al. Chronic liver injury induces conversion of biliary epithelial cells into hepatocytes. Cell Stem Cell 2018;23:114 ‐ 122.e113. 29937200 10.1016/j.stem.2018.05.022

[38] Wang EY , Yeh SH , Tsai TF , Huang HP , Jeng YM , Lin WH , et al. Depletion of β‐catenin from mature hepatocytes of mice promotes expansion of hepatic progenitor cells and tumor development. Proc Natl Acad Sci U S A 2011;108:18384‐18389. 22042854 10.1073/pnas.1116386108 PMC3215019

[39] Boulter L , Govaere O , Bird TG , Radulescu S , Ramachandran P , Pellicoro A , et al. Macrophage‐derived Wnt opposes Notch signaling to specify hepatic progenitor cell fate in chronic liver disease. Nat Med 2012;18:572‐579. 22388089 10.1038/nm.2667 PMC3364717

[40] Fabris L , Spirli C , Cadamuro M , Fiorotto R , Strazzabosco M . Emerging concepts in biliary repair and fibrosis. Am J Physiol Gastrointest Liver Physiol 2017;313:G102‐G116. 28526690 10.1152/ajpgi.00452.2016 PMC5582882

