# Supplementary material for: Hepatocyte‐Specific β‐Catenin Deletion During Severe Liver Injury Provokes Cholangiocytes to Differentiate Into Hepatocytes
Source: Hepatology. 2019 Jan 4;69(2):742–59. doi: 10.1002/hep.30270 (PMC6351199; doi:10.1002/hep.30270)
Supplement: Supplementary file 1 [file HEP-69-742-s001.pdf]

ONLINE SUPPLEMENT

Table of Content:

1. **Online Supplemental Methods**
2. **Online Table**
3. **Online Figures and Legends**

1. Online Supplemental Methods

Immunohistochemistry (IHC)

Tissue samples were drop-fixed in 10% buffered formalin for 48 hours prior to paraffin embedding. Samples were cut into 4 μm sections, deparaffinized, and washed with PBS. For antigen retrieval, samples were microwaved for 12 minutes in pH6 sodium citrate buffer (Cyclin D1, PanCK, GS, CD45) or Tris-EDTA buffer (Ki67), or were pressure cooked for 20 minutes in pH6 sodium citrate buffer (β-catenin), Dako Target Retrieval Solution (Dako, S1699) (CK19, EpCAM), or pH9 EDTA buffer (αSMA). After cooling, samples were placed in 3% H_2O_2 for 10 minutes to quench endogenous peroxide activity. After washing with PBS, slides were blocked with Super Block (ScyTek Laboratories, AAA500) for 10 minutes or 10% goat serum in PBS for 10 minutes (GS, p21). The primary antibodies were incubated at the following concentrations in antibody diluent (PBS + 1% BSA (Fisher BioReagents, BP1605-100) with 0.1% Tween™ 20 (Fisher BioReagents, BP337-500)): GS (Sigma G2781, 1:1500), Ki67 (Thermo Scientific RM-9106-S, 1:100), PanCK (Dako Z0622, 1:200), Cyclin D1 (Abcam ab134175, 1:200), β-catenin (Abcam ab32572, 1:100) for one hour at room temperature or at 4℃ overnight: p21 (Santa Cruz sc-471, 1:25), EpCAM (Biolegend 118201, 1:50), CK19 (DSHB TROMA III, 1:10). Samples were washed with PBS three times and incubated with the appropriate biotinylated secondary antibody (Vector Laboratories) diluted 1:500 or 1:1000 (GS) in antibody diluent for 30 minutes at room temperature. Samples were washed with PBS three times and sensitized with the Vectastain® ABC kit (Vector Laboratories, PK-6101) for 30 minutes. Following three washes with PBS color was developed with DAB Peroxidase Substrate Kit (Vector

Laboratories, SK-4100), followed by quenching in distilled water for five minutes. Slides were counterstained with hematoxylin (Thermo Scientific, 7211), dehydrated to xylene and coverslips applied with Cytoseal™ XYL (Thermo Scientific, 8312-4). For H&E staining, samples were deparaffinized and stained with hematoxylin (Thermo Scientific, 7211) and eosin (Thermo Scientific, 71204), followed by dehydration to xylene and application of a coverslip. For Sirius Red staining, samples were deparaffinized and incubated for one hour in Picro-Sirius Red Stain (American MasterTech, STPSRPT), washed twice in 0.5% acetic acid water, dehydrated to xylene, and coversliped. Images were taken on a Zeiss Axioskop 40 inverted brightfield microscope. Images for tiling were taken on a Zeiss Axio Observer.Z1 microscope and assembled utilizing ZEN Imaging software.

Immunofluorescence

Tissue samples were drop fixed in 10% buffered formalin overnight, cryopreserved in 30% sucrose in PBS overnight, frozen in OCT compound (Sakura, 4583) and stored at -80℃ or alternatively were paraffin embedded after formalin fixation. Cryopreserved samples were cut into 5 µm sections, allowed to air-dry, and then washed in PBS, while paraffin-embedded samples were cut into 4 µm sections and deparaffinized to PBS. Antigen retrieval was performed through pressure cooking for 20 minutes with Dako Target Retrieval Solution (Dako, S1699) or through microwaving in pH 6 sodium citrate buffer (PanCK, RFP, CYP2D6, GS, CK19). After cooling, slides were washed with PBS and permeabilized with 0.1% Triton X-100 in PBS for 20 minutes at room temperature. Samples were washed three times with PBS and then blocked with 2% Donkey serum in 0.1% Tween™ 20 in PBS (antibody diluent) for 30 minutes at room temperature.

Antibodies were diluted as follows: β-catenin (Abcam ab32572, 1:100), PanCK (Dako Z0622, 1:200), Hnf4α (Santa Cruz sc-6556, 1:50), GFP (Abcam ab13970, 1:200), CK19 (DSHB TROMA-III-s, 29 μg/ml), PCNA (Santa Cruz sc-56, 1:1000), RFP (Rockland 600-401-379, 1:200), GS (Abcam ab73593, 1/200), CYP2D6 (Gift from R. Wolfe, University of Dundee, 1/500) in antibody diluent and incubated at 4℃ overnight. Samples were washed three times in PBS and incubated with the proper fluorescent secondary antibody (AlexaFluor 488/555/647, Invitrogen) diluted 1:800 in antibody diluent for two hours at room temperature. Samples were washed three times with PBS and incubated with DAPI (Sigma, B2883) for 1 minute. Samples were washed three times with PBS and mounted with fluromount (SouthernBiotech) or ProLong™ Gold antifade reagent (Invitrogen, P10144). Images were taken on a Nikon Eclipse Ti epifluorescence microscope or a Zeiss LSM700 confocal microscope.

Western blotting

To extract protein, whole liver tissue was homogenized in RIPA buffer as previously described [28]. Protein was separated on pre-cast 4-20% or 7.5% polyacrylamide gels (Bio-Rad) and transferred to a nitrocellulose membrane using the Trans-Blot Turbo Transfer System (Bio-Rad). Membranes were blocked for 30 minutes with 5% skim milk (LabScientific, Cat# M0841) or 5% BSA in Blotto buffer (0.15 M NaCl, 0.02 M Tris pH 7.5, 0.1% Tween in dH2O), and incubated with primary antibodies at 4℃ overnight at the following concentrations: β-catenin (BD Biosciences 610154, 1:1000 in 5% milk), Active β-catenin (Cell Signaling cs-4270, 1:800 in 5% BSA), Cyclin D1 (Thermo Fisher RB-9041-P, 1:200 in 5% milk), GS (Santa Cruz sc-74430, 1:2000 in 5% milk), p21 (Santa Cruz sc-271532, 1:50 in 5% milk), αSMA (Abcam ab5694, 1:1000 in 5% milk),

GAPDH (Santa Cruz sc-25778, 1:1000 in 5% milk). Membranes were washed in Blotto buffer and incubated with the appropriate HRP-conjugated secondary antibody for 1 – 3 hours at room temperature. Membranes were washed with Blotto buffer, and bands were developed utilizing SuperSignal® West Pico Chemiluminescent Substrate (Thermo Scientific, Prod# 34080) and visualized by autoradiography.

RT-PCR

Whole liver was homogenized in TRIzol™ (Thermo Scientific, Cat# 15596026), treated with chloroform, and nucleic acid was precipitated with isopropanol. Cellular DNA was digested with DNA-free™ Kit (ambion, AM1906), and RNA was reverse-transcribed into cDNA using SuperScript® III (Invitrogen, 18080-044). Real-time PCR was performed in technical triplicate on a StepOnePlus™ Real-Time PCR System (Applied Biosystems, Cat# 4376600) using the Power SYBR® Green PCR Master Mix (Applied Biosystems, 4367660). Target gene expression was normalized to the average of two housekeeping genes (*Gapdh* and *Rn18s*), and fold change was calculated utilizing the ΔΔ-Ct method. Primers are listed in Table 1.

2. Online Table

Table 1: Sequence of primers used in the study.

Gene	Forward Primer (5' – 3')	Reverse Primer (5' – 3')
Ccnd1	TTTCTTTCCAGAGTCATCAAGTGT	TGACTCCAGAAGGGCTTCAA
Ctnnb1	ACTTGCCACACGTGCAATTC	AAGGTTGTGCAGAGTCCCAG
Col1a1	TCCGGCTCCTGCTCCTCTTA	GTATGCAGCTGACTTCAGGGATGT
Acta2	CCGAGATCTCACCGACTACC	TCCAGAGCGACATAGCACAG
Sox9	GTGCAAGCTGGCAAAGTTGA	TGCTCAGTTCACCGATGTCC
Krt19	CCAGGAAGCCCACTACAACAA	TCGAGGGAGGGGTTAGAGTAAA
Gapdh	AACTTTGGCATTGTGGAAGG	ACACATTGGGGGTAGGAACA
Rn18s	GTAACCCGTTGAACCCCATT	CCATCCAATCGGTAGTAGCG

3. Online Figures and Legends

Figure S1:

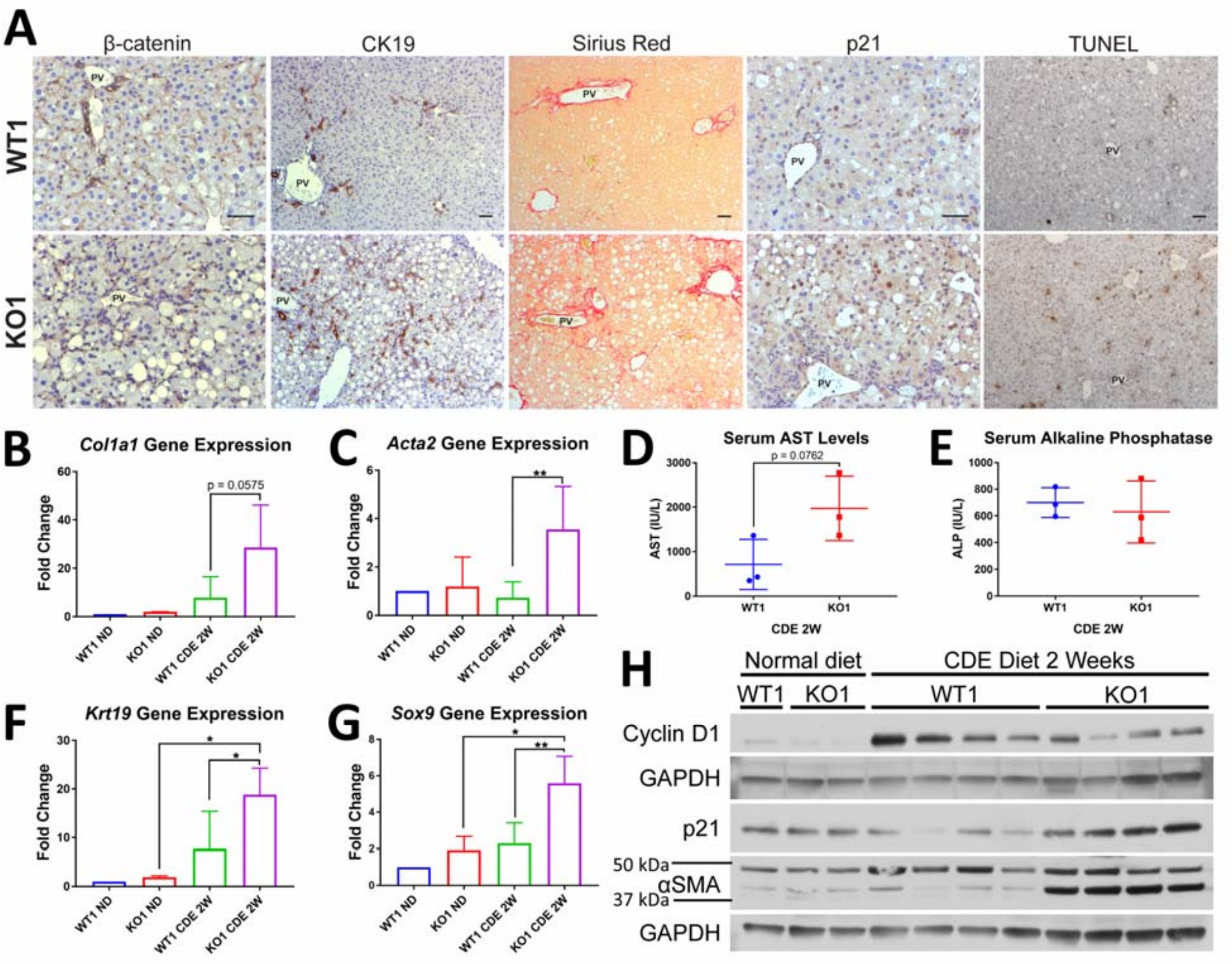

Figure S1: KO1 mice develop fibrosis and a robust BEC response after two weeks of CDE diet.

(A) IHC staining reveals loss of β-catenin expression in both hepatocytes and BECs in KO1 mice. Additionally, KO1 mice display robust expansion of BECs by CK19 staining, fibrosis by Sirius Red, an increase in p21-positive hepatocytes compared to WT1 mice, and TUNEL-positive cells in both WT1 and KO1 mice after two weeks of CDE diet (scale bar 50 μm). PV = portal vein.

(B) KO1 mice display an increase in *Col1a1* gene expression (one-way ANOVA, p =

0.0575).

(C) KO1 mice display a significant increase in *Acta2* (α-smooth muscle actin) gene expression compared to WT1 mice (one-way ANOVA, $p < 0.01$).

(D) Elevated serum AST levels in KO1 mice compared to WT1 mice after two weeks of CDE diet (t-test, $p = 0.0762$).

(E) No difference in serum ALP levels in KO1 and WT1 mice after two weeks of CDE diet.

(F) KO1 mice display a significant increase in *Krt19* gene expression compared to WT1 mice (one-way ANOVA, $p < 0.05$) and KO1 mice on normal diet (one-way ANOVA, $p < 0.05$).

(G) KO1 mice display a significant increase in *Sox9* gene expression compared to WT1 mice (one-way ANOVA, $p < 0.01$) and KO2 mice on normal diet (one-way ANOVA, $p < 0.05$).

(H) KO1 mice on CDE diet for two weeks express less Cyclin D1, more p21, and more α-smooth muscle actin (molecular weight 42 kDa) compared to WT1 littermates.

Figure S2:

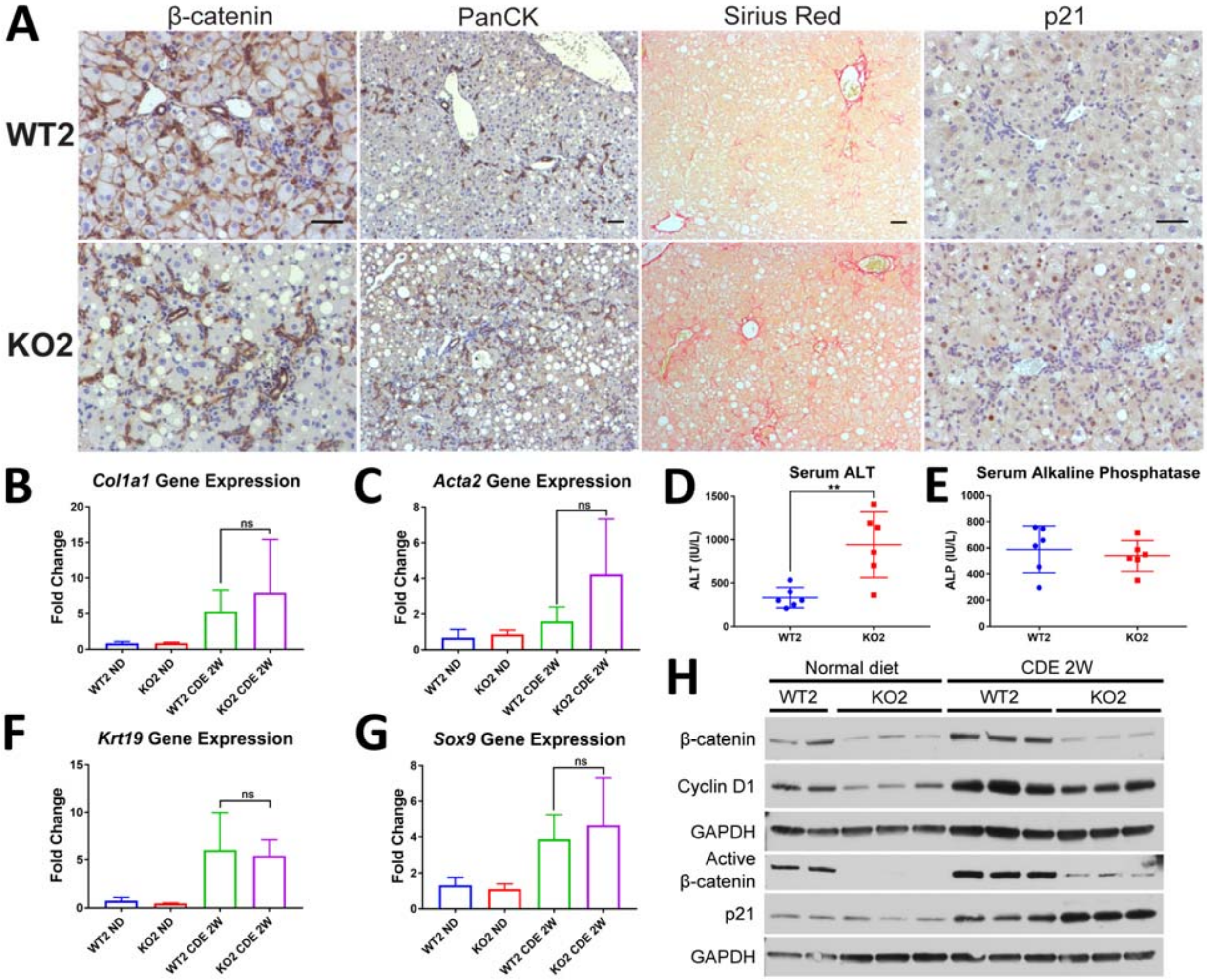

Figure S2: KO2 mice develop fibrosis and a robust BEC response after two weeks of CDE diet.

(A) IHC staining reveals a lack of β-catenin expression in hepatocytes in KO2 mice. Additionally, Pan-cytokeratin staining reveals a robust BEC response, Sirius Red the development of fibrosis, and p21 staining reveals an increase in p21-positive hepatocytes in KO2 mice after two weeks of CDE diet compared to WT2 mice (scale bar 50 μm).

(B) WT2 and KO2 mice display an increase in *Col1a1* gene expression of two weeks of CDE diet.

(C) WT2 and KO2 mice display an increase in *Acta2* (α-smooth muscle actin) gene expression after two weeks of CDE diet.

(D) Significantly elevated serum ALT levels in KO2 mice after two weeks of CDE diet (t-test, $p < 0.01$).

(E) No difference in serum ALP levels in WT2 and KO2 mice after two weeks of CDE diet.

(F) WT2 and KO2 mice display an increase in *Krt19* gene expression after two weeks of CDE diet.

(G) WT2 and KO2 mice display an increase in *Sox9* gene expression after two weeks of CDE diet.

(H) KO2 mice do not display an increase in total β-catenin levels but do show an increase in active β-catenin after two weeks of CDE diet compared to KO2 mice on normal diet. KO2 mice also display less Cyclin D1 and more p21 expression compared to WT2 mice after two weeks of CDE diet.

Figure S3:

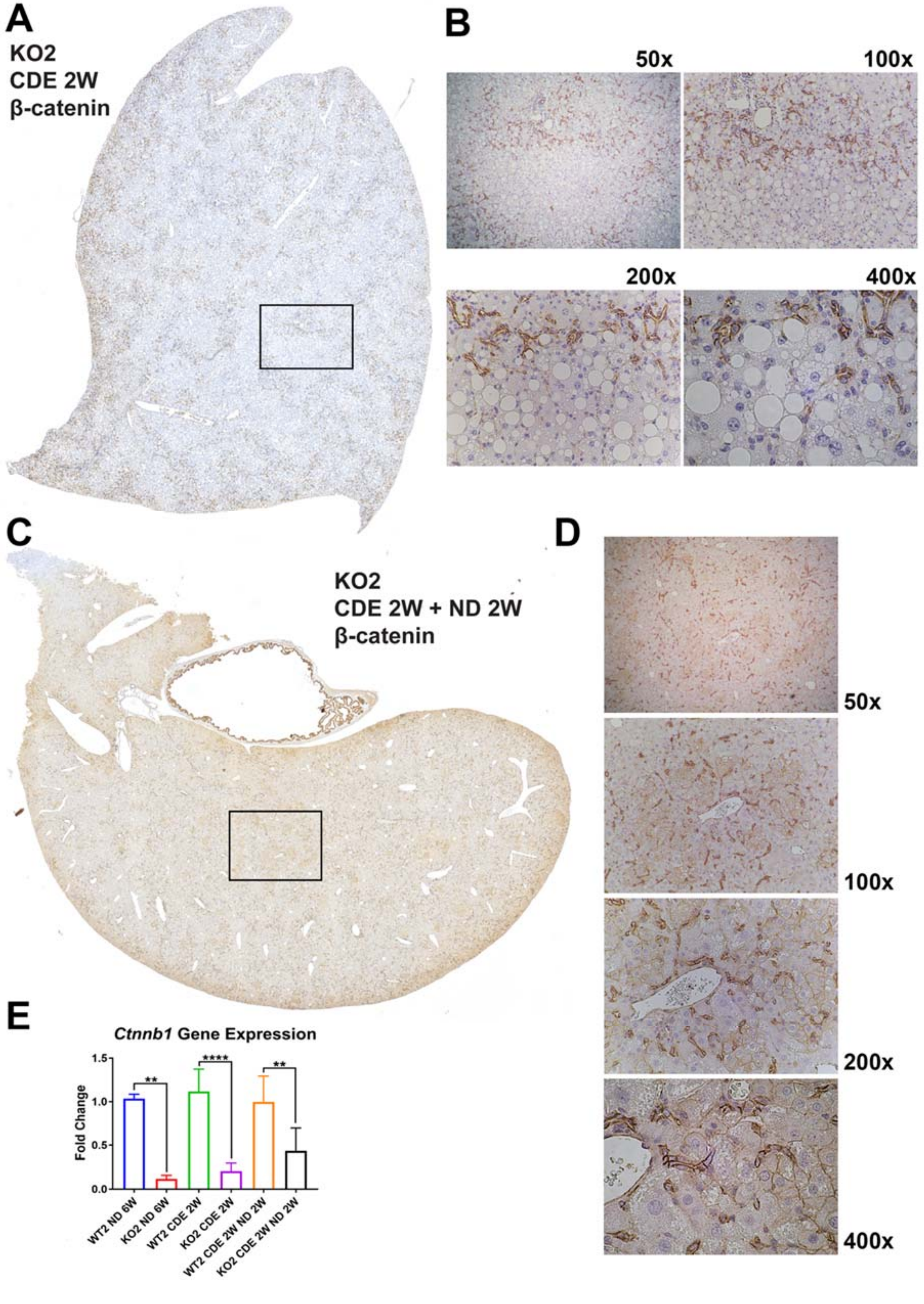
A
KO2
CDE 2W
β-catenin
B
50x
100x
200x
400x
C
KO2
CDE 2W + ND 2W
β-catenin
D
50x
100x
200x
400x
E
Ctnnb1 Gene Expression
Fold Change
1.5
1.0
0.5
0.0
**
****
**
WT2 ND 6W
KO2 ND 6W
WT2 CDE 2W
KO2 CDE 2W
WT2 CDE 2W ND 2W
KO2 CDE 2W ND 2W

Figure S3: Expansion of β-catenin-positive hepatocytes occurs only after recovery on normal diet.

(A) Tiled β-catenin IHC staining of an entire lobe from the liver of a KO2 mouse after 2 weeks of CDE diet reveals dramatic expansion of β-catenin-positive BECs, but no clusters of β-catenin-positive hepatocytes.

(B) Serial magnifications of a representative area from Figure S3A (open box) verifies dramatic expansion of β-catenin-positive BECs, but no clusters of β-catenin-positive hepatocytes (50x, 100x, 200x, 400x).

(C) Tiled β-catenin IHC staining of an entire lobe from the liver of a KO2 mouse after 2 weeks of CDE diet followed by 2 weeks of recovery on normal diet reveals clusters of β-catenin-positive hepatocytes appearing across the entire lobe.

(D) Serial magnifications of a representative area from Figure S3C (open box) verifies clusters of β-catenin-positive hepatocytes appearing in close association with ductules (50x, 100x, 200x, 400x).

(E) Expression of *Ctnnb1* is dramatically reduced in KO2 mice compared to WT2 mice on normal diet for 6 weeks. After 2 weeks of CDE diet followed by 2 weeks of recovery on normal diet, expression of *Ctnnb1* is trending upward in KO2 mice, although it is still significantly reduced compared to WT2 levels (one-way ANOVA, ** = $p < 0.01$. **** = $p < 0.0001$).

Figure S4:

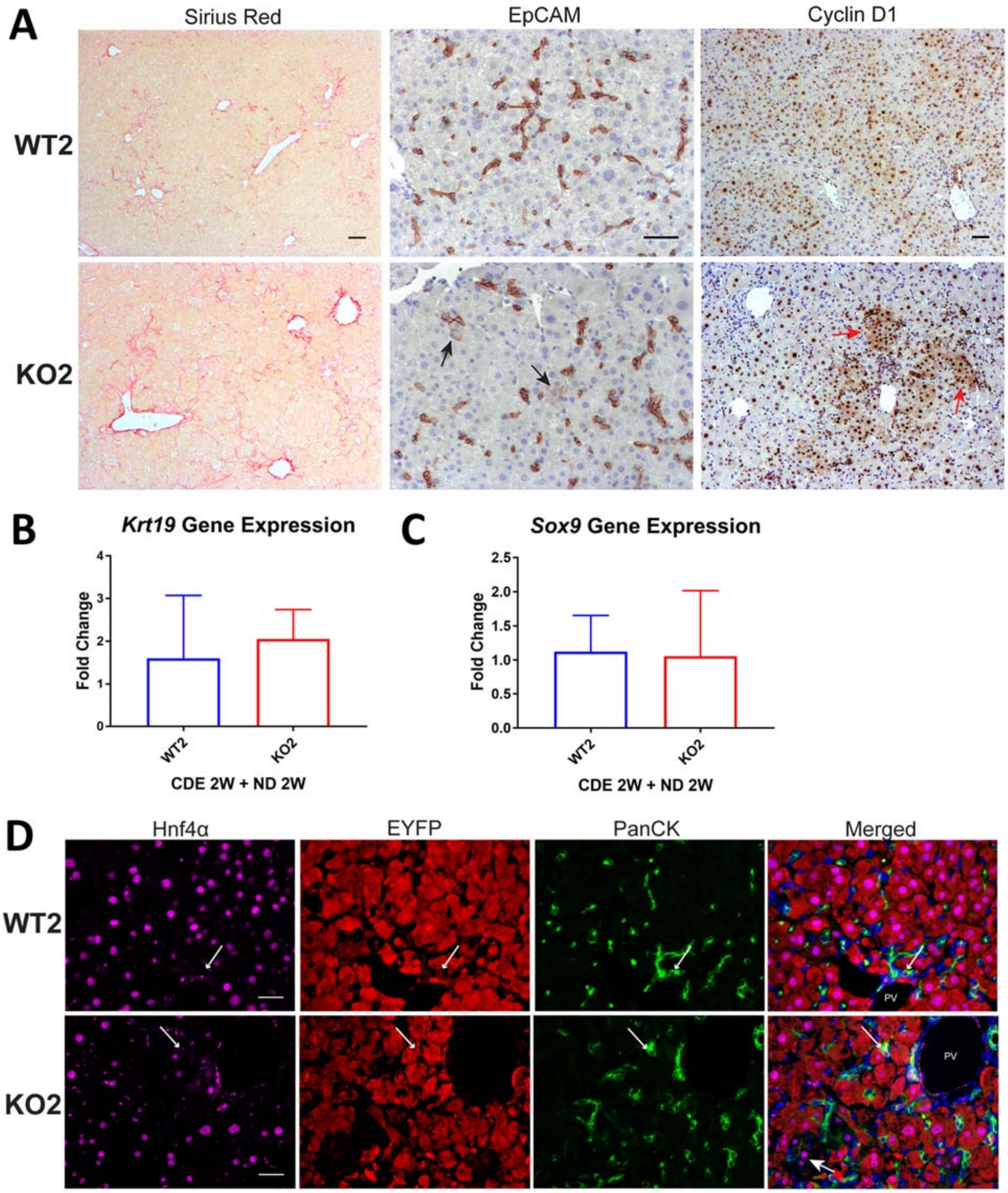

Figure S4: Robust BEC response in WT2 and KO2 mice after CDE diet and

recovery.

(A) There is sustained fibrosis and a continued BEC response in both WT2 and KO2 mice after CDE diet and recovery. Rare EpCAM-positive cells with hepatocyte morphology are detectable in KO2 mice (black arrows). Additionally, clusters of presumable BEC-derived hepatocytes (red arrows) are strongly Cyclin D1 positive in KO2 mice (scale bar 50 μm).

(B) The level of *Krt19* gene expression is comparable between WT2 and KO2 mice after 2 weeks of CDE diet followed by 2 weeks of recovery on normal diet.

(C) The level of *Sox9* gene expression is comparable between WT2 and KO2 mice after 2 weeks of CDE diet followed by 2 weeks of recovery on normal diet.

(D) The majority of BECs are negative for EYFP in WT2 and KO2 mice after CDE diet and recovery. However, rare EYFP cells positive for BEC markers are detectable in both WT2 and KO2 mice, potentially suggesting hepatocyte-to-BEC transdifferentiation (scale bar 50 μm). PV = portal vein.

Figure S5:

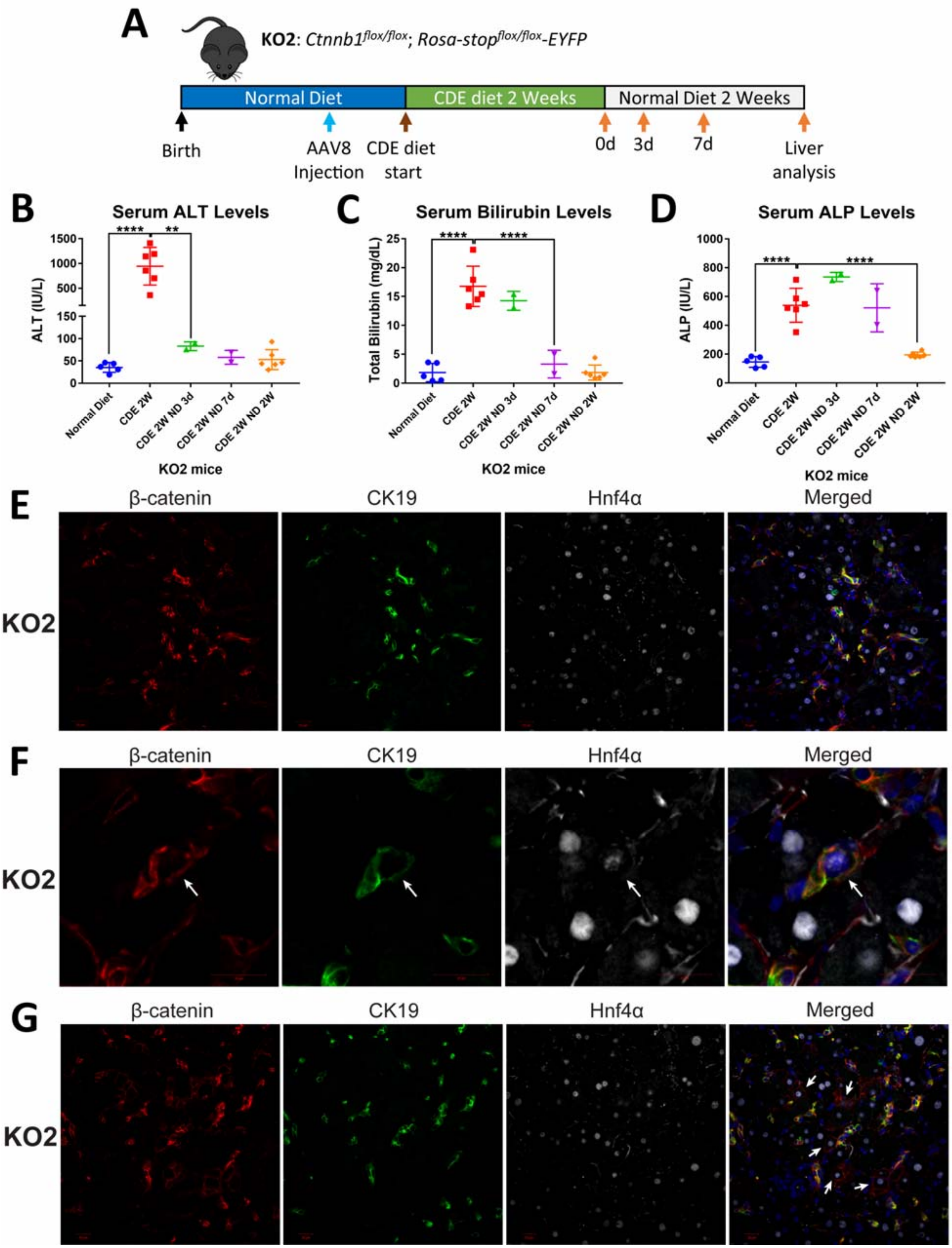
A
KO2: Ctnnb1flox/flox; Rosa-stopflox/flox-EYFP
Normal Diet
CDE diet 2 Weeks
Normal Diet 2 Weeks
Birth
AAV8 Injection
CDE diet start
0d
3d
7d
Liver analysis
B
Serum ALT Levels
ALT (IU/L)
C
Serum Bilirubin Levels
Total Bilirubin (mg/dL)
D
Serum ALP Levels
ALP (IU/L)
Normal Diet
CDE 2W
CDE 2W ND 3d
CDE 2W ND 7d
CDE 2W ND 2W
KO2 mice
E
F
G
β-catenin
CK19
Hnf4α
Merged
KO2

Figure S5: Expansion of BEC-derived, β-catenin-positive hepatocytes during early recovery on normal diet.

(A) Schematic of KO2 mice placed on normal diet for two weeks and then allowed to recovery on normal diet for either 0, 3, 7, or 14 days.

(B) Serum ALT levels are elevated in KO2 mice on CDE diet for 2 weeks, but are dramatically reduced as early as 3 days after recovery on normal diet (One-way ANOVA, ** $p < 0.01$, **** $p < 0.0001$).

(C) Serum bilirubin levels are elevated in KO2 mice on CDE diet for 2 weeks, and only begin to show a reduction after 7 days of recovery on normal diet (One-way ANOVA, **** $p < 0.0001$).

(D) Serum ALP levels are elevated in KO2 mice on CDE diet for 2 weeks and begin to show a reduction after two weeks of recovery on normal diet (One-way ANOVA, **** $p < 0.0001$).

(E) After 2 weeks of CDE diet, β-catenin-positive cells are positive for BEC-marker CK19 but negative for Hnf4α (scale bar 20 μm).

(F) As early as 3 days of recovery following CDE diet-induced liver injury, β-catenin-positive cells which express CK19 and weakly express Hnf4α (white arrow) can be detected (scale bar 20 μm).

(G) After 7 days of recovery on normal diet following CDE diet-induced liver injury, there are clusters containing multiple β-catenin-positive, Hnf4α-positive hepatocytes (white arrows, scale bar 20 μm).

Figure S6:

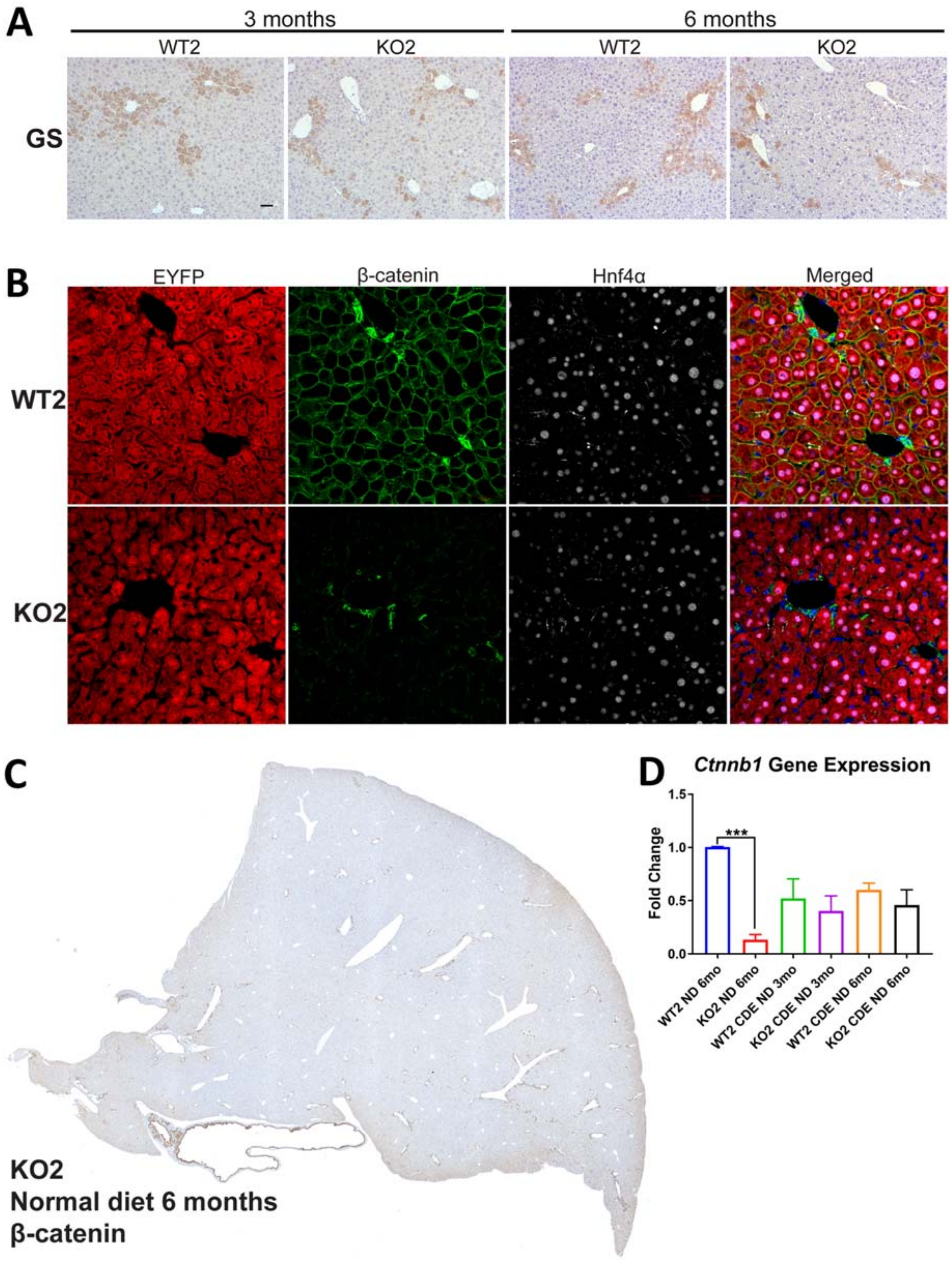
A
3 months
6 months
WT2
KO2
WT2
KO2
GS
B
EYFP
β-catenin
Hnf4α
Merged
WT2
KO2
C
KO2
Normal diet 6 months
β-catenin
D
Ctnnb1 Gene Expression
Fold Change
1.5
1.0
0.5
0.0
***
WT2 ND 6mo
KO2 ND 6mo
WT2 CDE ND 3mo
KO2 CDE ND 3mo
WT2 CDE ND 6mo
KO2 CDE ND 6mo

Figure S6: Expansion of EYFP-negative hepatocytes occurs only after exposure to CDE diet in KO2 mice.

(A) Expression of GS partially reappears in KO2 mice after CDE diet-induced liver injury and either 3 or 6 months of recovery on normal diet.

(B) Virtually all hepatocytes are EYFP-positive in both control WT2 and KO2 mice left on normal diet for 6 months (scale bar 50 μm).

(C) Tiled IHC β-catenin staining of a lobe from a KO2 mouse left on normal diet for 6 months after AAV8 injection reveals virtually no β-catenin-positive hepatocytes.

(D) There is dramatically reduced hepatic *Ctnnb1* expression in control KO2 mice left on normal diet for 6 months compared to WT2 controls. However, in KO2 mice placed on CDE diet followed by up to 6 months of recovery on normal diet the level of hepatic *Ctnnb1* expression is approaching WT2 levels (one-way ANOVA, *** = $p < 0.001$).

Figure S7:

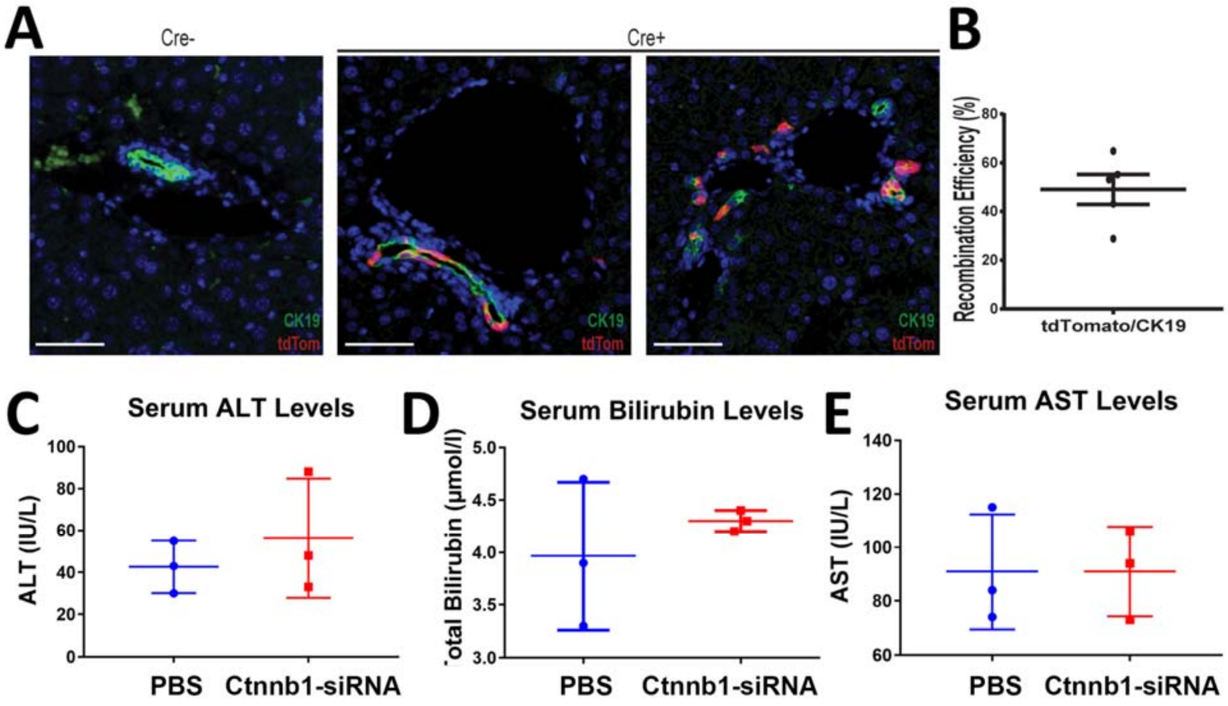

Figure S7: BEC-derived hepatocytes begin to repopulate the liver in Ctnnb1-siRNA-injected mice.

(A) After tamoxifen administration, only CK19-positive BECs are labeled with tdTomato. Expression of tdTomato is not detected in mice that do not express Cre recombinase (scale bar 100 μm).

(B) Quantification of recombination efficiency of CK19-positive cells following tamoxifen injections.

(C) Serum ALT levels are normalizing in Ctnnb1-siRNA-injected mice after CDE diet and recovery.

(D) Serum Bilirubin levels are normalizing in Ctnnb1-siRNA-injected mice after CDE diet and recovery.

(E) Serum AST levels are normalizing in Ctnnb1-siRNA-injected mice after CDE diet

and recovery.
